# Non-growth substrate ethane perturbs core methanotrophy in obligate methanotroph *Methylosinus trichosporium* OB3b upon nutrient availability

**DOI:** 10.1128/aem.00969-25

**Published:** 2025-07-10

**Authors:** Sunho Park, Chungheon Shin, Craig S. Criddle, Jaewook Myung

**Affiliations:** 1 Department of Civil and Environmental Engineering, Korea Advanced Institute of Science and Technology (KAIST) 34968 , Daejeon, Republic of Korea; 2 Department of Civil and Environmental Engineering, Stanford University 198901, Stanford, California, USA; 3 Codiga Resource Recovery Center, Stanford University 6429, Stanford, California, USA; University of Delaware, Lewes, Delaware, USA

**Keywords:** methanotrophs, ethane, cometabolism, non-growth substrate, nutrient availability, polyhydroxybutyrate

## Abstract

Obligate methanotrophs grow exclusively on C1 compounds; however, their widespread ecological distribution and ability to produce polyhydroxybutyrate (PHB) under nutrient stress suggest a greater-than-expected degree of metabolic flexibility. However, the metabolic responses of type II obligate methanotrophs to heterogeneous substrate conditions, common in natural and engineered environments, remain poorly understood. Here, we investigated how ethane (C_2_H_6_), a non-growth substrate and methane monooxygenase (MMO) co-substrate, affects the metabolism of *Methylosinus trichosporium* OB3b under varying C_2_H_6_ concentrations and nutrient availabilities (CH_4_, O_2_, and nitrogen). Exposure to C_2_H_6_ impaired CH_4_ oxidation and cell growth while stimulating PHB synthesis, with these effects becoming more pronounced at higher C_2_H_6_ levels. Externally supplemented reducing equivalents were preferentially directed toward C_2_H_6_ uptake rather than CH_4_ oxidation under co-substrate conditions. Additionally, acetate derived from C_2_H_6_ exerted divergent effects on carbon assimilation during growth and PHB accumulation phases, without directly interfering with CH_4_ oxidation. RT-qPCR unraveled that MMO gene expression responded to C_2_H_6_ in a nutrient availability-dependent manner, with opposing patterns during growth and PHB accumulation phases. Interestingly, MMO transcript levels did not correlate with CH_4_ oxidation rates, suggesting that MMO mRNA abundance may not serve as a reliable proxy for actual methanotrophic activity under co-substrate conditions. These findings highlight the significant effects of heterogeneous substrate environments—containing co-metabolic substrates and microbial metabolites—on obligate methanotrophs, offering insights for revisiting methanotrophic activities in ecosystems and optimizing biotechnological applications.

**IMPORTANCE:**

Type II methanotrophs present a dual advantage: mitigating methane emissions and producing bioproducts such as polyhydroxybutyrate (PHB). However, their full potential remains untapped, partly due to a limited understanding of how co-occurring gases influence their metabolism. Methane-rich emissions from both natural and anthropogenic sources are frequently accompanied by secondary gases, such as ethane, which create heterogeneous substrate conditions. This study reveals that ethane, a non-growth co-metabolic substrate, significantly modulates the metabolism of type II obligate methanotrophs, affecting microbial growth, methane oxidation, and PHB synthesis. These results advance our understanding of the metabolic plasticity of these organisms and also reveal new opportunities to leverage secondary substrates for selectively fine-tuning beneficial methanotrophic activities, such as biopolymer production.

## INTRODUCTION

Methane (CH_4_), a potent greenhouse gas, has a 20-year timescale global warming potential (GWP_20_) more than 84 times greater than that of carbon dioxide (CO_2_) (1). It is emitted from both natural (e.g*.,* wetlands, termites, and oceans) and anthropogenic activities (e.g*.,* agriculture, fossil fuels, and landfills) (1–3). Aerobic methane-oxidizing bacteria, or methanotrophs, play a pivotal role in the global CH_4_ cycle as a primary biological sink for atmospheric CH_4_ (2, 4). Beyond their environmental significance, type II methanotrophs are of particular interest due to their ability to synthesize polyhydroxybutyrate (PHB), a biodegradable polymer, positioning them as a promising platform for value-added bioconversion of CH_4_. To advance methanotroph-based bioprocesses, it is crucial to deepen our understanding of their physiology across diverse environmental conditions.

In nature, methanotrophs are frequently exposed to fluctuating substrate conditions and often tailor their metabolic flexibility to match the resource availability (5, 6). Such adaptations include the use of alternative electron acceptors and donors (7–14), fermentation under O_2_ limitation (13, 15), nitrogen fixation (16–18), co-metabolism (19, 20), and facultative methanotrophy exhibiting growth on multi-carbon compounds such as simple organic acids, alcohols, and short-chain alkane gases (21–27). These versatile metabolic traits likely explain their ubiquitous distribution across a range of ecological niches (13, 14).

Despite decades of research, significant gaps remain in our understanding of how aerobic methanotrophy functions in the presence of heterogeneous substrates, where the primary substrate (CH_4_) coexists with secondary substrates, or under variable CH_4_ and O_2_ availability (28). Moreover, most studies on metabolic versatility have largely focused on growth potential, with a limited investigation into its relevance to PHB synthesis—a survival strategy under nutrient-imbalanced conditions in type II methanotrophs (29). Given that microbial metabolism is regulated both at the substrate entry level and in central metabolic pathways (30, 31), the introduction of miscellaneous substrates—particularly those pertaining to the central metabolism of methanotrophs—can exert a significant impact on methanotrophy and PHB synthesis.

Ethane (C_2_H_6_), the second most abundant component of natural gas, reaching up to 15% after CH_4_ (32–34), is commonly found in methanotroph habitats (e.g*.,* natural gas exploitation, crude oil refining [35, 36], hydrothermal vent fluids [37, 38], and deep marine sediments [39, 40]). Given that C_2_H_6_ can be oxidized to ethanol by methane monooxygenase (MMO), the key enzyme for CH_4_ oxidation in all aerobic methanotrophs (28, 41–43), its presence may substantially impact methanotrophic metabolism when CH_4_ and C_2_H_6_ coexist. Unlike other widely studied co-metabolic compounds, such as chlorinated hydrocarbons, which simply deplete reducing power generated from CH_4_ oxidation and thereby inhibit CH_4_ consumption (44–47), the oxidation of C_2_H_6_ can release net electrons, suggesting potentially distinct and more nuanced co-metabolic interactions. Additionally, in our previous study, type II obligate methanotroph *Methylocystis parvus* OBBP was able to produce PHB using C_2_H_6_ in the absence of CH_4_ (32).

**Fig 1:**
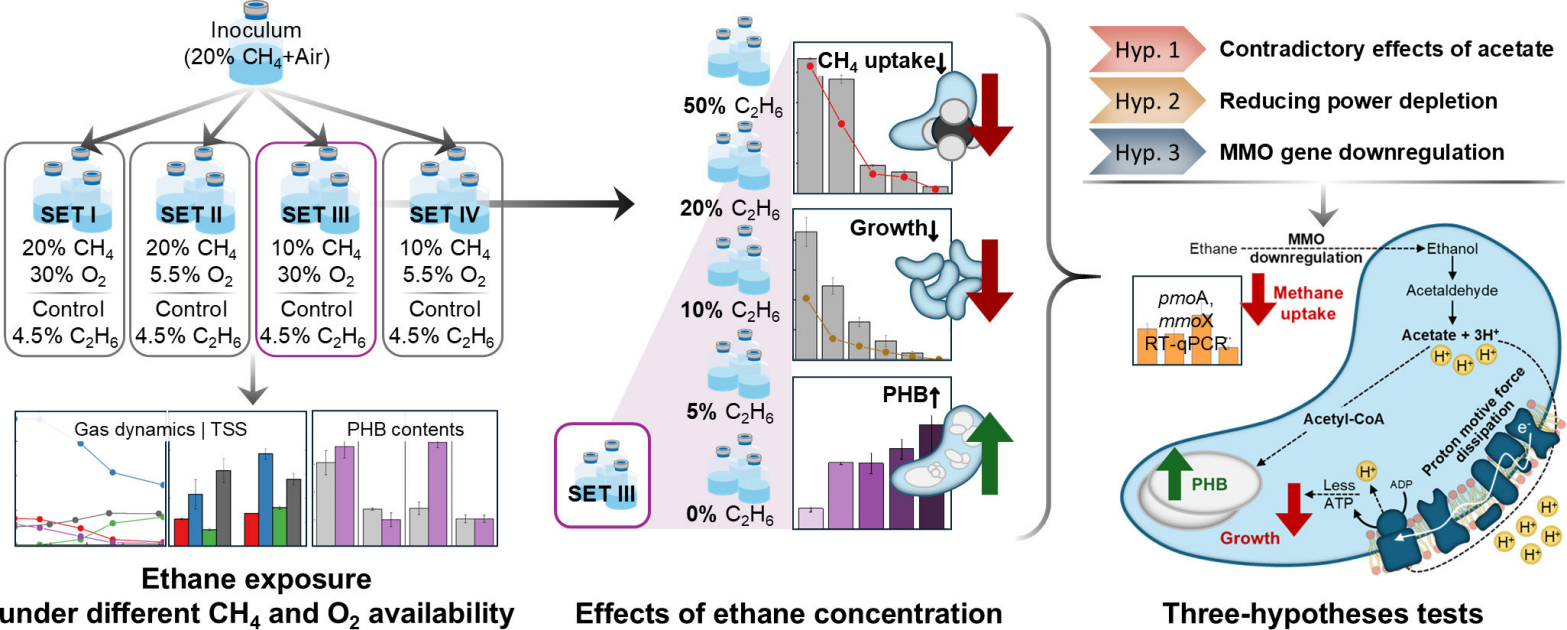
Sequential experimental setups for investigating *M. trichosporium* OB3b responses. The addition of 4.5% C_2_H_6_ was primarily tested across four distinct sets (SET I–IV) with varying CH_4_ and/or O_2_ availabilities. Subsequently, five different concentrations of C_2_H_6_ were tested under the selected set. Detailed follow-up hypotheses tests were conducted to elucidate the results from the preceding steps. All experiments were carried out in triplicate serum bottles.

To this end, we designed a series of experiments using *Methylosinus trichosporium* OB3b, a type II obligate methanotroph, under varying substrate conditions (Fig. 1). First, we examined the growth and PHB synthesis in the presence or absence of C_2_H_6_ across four distinct experimental sets with replete or limited CH_4_ and/or O_2_. Second, we explored the effects of different concentrations of C_2_H_6_. To further probe the metabolic mechanisms underlying C_2_H_6_-driven responses, subsequent experiments with methanol, formate, and acetate supplementation, along with RT-qPCR analysis, were conducted. Our results reveal that C_2_H_6_ significantly influences type II obligate methanotrophy—not through altering *pmoA* transcription but primarily by scavenging reducing power and generating key metabolic intermediates—offering new insights into the metabolic interplay between coexisting short-chain alkanes.

## RESULTS AND DISCUSSION

### Effects of C_2_H_6_ addition under varying CH_4_ and/or O_2_ availabilities

**Fig 2:**
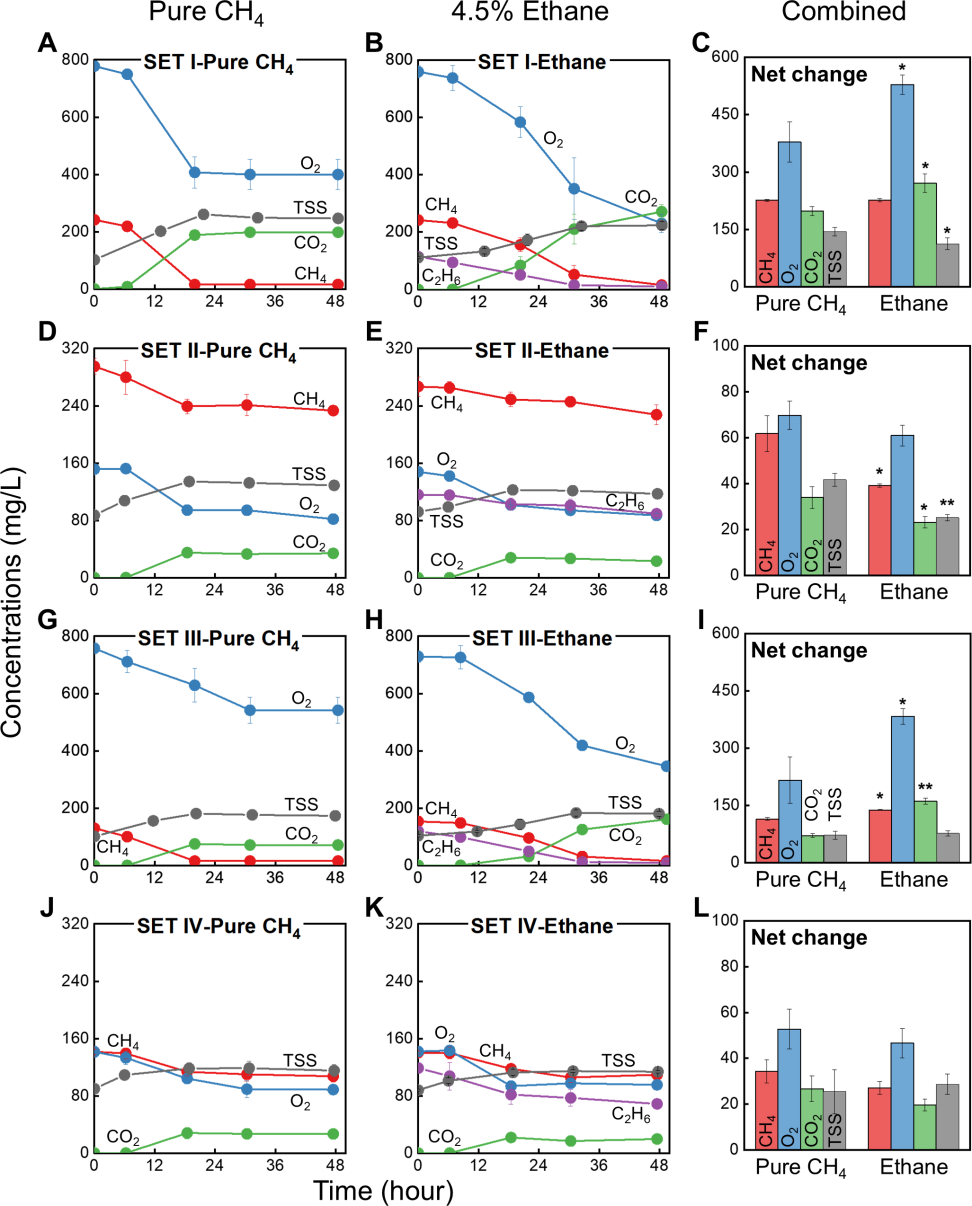
Activity of *M. trichosporium* OB3b cultures during 48 h growth phases under four different gas conditions, with CH_4_ (Pure CH_4_, left panels) or CH_4_ plus C_2_H_6_ (Ethane, middle panels). Consumption of CH_4_ (red), O_2_ (blue), and C_2_H_6_ (purple), as well as production of CO_2_ (green) and total suspended solids (TSS, gray), are shown. Experimental sets: SET I (20% CH_4_, 30% O_2_ **[A–C]**), SET II (20% CH_4_, 5.5% O_2_ **[D–F]**), SET III (10% CH_4_, 30% O_2_ **[G–I]**), and SET IV (10% CH_4_, 5.5% O_2_ **[J–L]**). Net changes in CH_4_ and O_2_ (consumed), and CO_2_ and TSS (produced), during the 48 h cultivation are summarized in the right panels (**C, F, I, L**). Single asterisk (*) and double asterisks (**) indicate significant differences between the C_2_H_6_ treatments and pure CH_4_ controls (*P*-value < 0.05 and < 0.005, respectively). Y-axis concentrations represent nominal aqueous-phase concentrations, obtained by dividing the total mass per bottle by the liquid-phase volume (0.05 L). All experiments were carried out in triplicate, and data are reported as mean ± standard deviation (SD) (File S1).

The pure culture of *M. trichosporium* OB3b was cultivated under four different CH_4_ and/or O_2_-abundant/insufficient conditions, each comprising two sub-conditions (a 4.5% C_2_H_6_-supplemented condition and a CH_4_-only control). All gaseous components were periodically monitored during the 48 h nutrient-balanced growth phase (Fig. 2; File S1).

In all C_2_H_6_-supplemented treatments across the four sets, C_2_H_6_ was simultaneously consumed with CH_4_ during the growth of *M. trichosporium* OB3b, even under O_2_-limited SET II and IV (Fig. 2B, E, H and K). Notably, although our previous study reported neither growth nor PHB accumulation when C_2_H_6_ was supplied as the sole carbon source (32), the present study revealed C_2_H_6_ oxidation when co-fed with CH_4_. This suggests that *M. trichosporium* OB3b can co-metabolize C_2_H_6_—a process where non-growth substrates are degraded in the presence of primary growth substrates (44, 48)—and that C_2_H_6_ oxidation likely requires concurrent CH_4_ oxidation.

In *M. trichosporium* OB3b, both CH_4_ and C_2_H_6_ oxidation require two reducing equivalents, such as NADH (equations 1 and 2) (28, 32, 49, 50). After CH_4_ activation by MMO (equation 1), methanol is sequentially oxidized to formaldehyde, formate, and CO_2_ (equation 3), regenerating reducing equivalents required to sustain MMO activity (50). Although two electrons are consumed for MMO activation, the remaining four electrons (equation 4) are shuttled through the electron transport chain, ultimately reducing O_2_ and generating ATP via oxidative phosphorylation (51, 52). Conversely, C_2_H_6_ oxidation yields acetate and provides only two net electrons (equations 5 and 6), contributing less reducing power compared with CH_4_ oxidation. Therefore, the dependence of C_2_H_6_ oxidation on CH_4_ availability can be partly attributed to the need for continuous replenishment of reducing equivalents, derived from CH_4_ oxidation.


(eq. 1) $$CH_{4}+O_{2}+2H^{+}+2e^{-}\to CH_{3}OH+H_{2}O$$

(eq. 2) $$C_{2}H_{6}+O_{2}+2H^{+}+2e^{-}\to C_{2}H_{5}OH+H_{2}O$$

(eq. 3) $$CH_{3}OH+H_{2}O\to CO_{2}+6H^{+}+6e^{-}$$

(eq. 4) $$CH_{4}+O_{2}\to CO_{2}+4H^{+}+4e^{-}$$

(eq. 5) $$C_{2}H_{5}OH+H_{2}O\to CH_{3}COO^{-}+5H^{+}+4e^{-}$$

(eq. 6) $$C_{2}H_{6}+O_{2}\to CH_{3}COO^{-}+3H^{+}+2e^{-}$$

In O_2_-sufficient SET I and III, C_2_H_6_ was completely depleted, and its addition resulted in significantly increased O_2_ consumption and CO_2_ production compared with the CH_4_-only controls (Fig. 2C and I). In theory, 1 mole of O_2_ is required to activate each mole of CH_4_ or C_2_H_6_ via MMO (equations 1and 2and 2). Thus, the simultaneous supply of CH_4_ and C_2_H_6_ heightened O_2_ demand, yielding a higher O_2_-to-CH_4_ consumption ratio in the C_2_H_6_ treatments (2.34 ± 0.12 and 2.78 ± 0.13 in SET I and III, respectively) compared with the controls (1.68 ± 0.25 and 1.89 ± 0.55 in SET I and III, respectively).

Interestingly, the addition of C_2_H_6_ markedly suppressed the maximum specific CH_4_ utilization rate (*q_max_CH4_*), particularly in SET I, II, and III, compared with the pure CH_4_ controls (Table 1, *P*-value < 0.01). This reduction may originate from competition for reducing equivalents (equations 1 and 2), which are diverted between CH_4_ and C_2_H_6_ oxidation. This hypothesis will be further addressed in the subsequent section.

**TABLE 1:** Kinetic parameters of *M. trichosporium* OB3b cultivated under four different sets, with or without 4.5% C_2_H_6_ (vol/vol) during the nutrient-balanced growth^*a*^^,^^*b*^^,^^*c*^

Sample codes	SET I (20% CH_4_, 30% O_2_)		SET II (20% CH_4_, 5.5% O_2_)		SET III (10% CH_4_, 30% O_2_)		SET IV (10% CH_4_, 5.5% O_2_)	
	Pure CH_4_	Ethane	Pure CH_4_	Ethane	Pure CH_4_	Ethane	Pure CH_4_	Ethane
*q_max_CH4_* (mg CH_4_·mg TSS^−1^·h^−1^)	0.063 ± 0.002	0.041 ± 0.003**	0.029 ± 0.003	0.010 ± 0.002**	0.036 ± 0.002	0.028 ± 0.002*	0.013 ± 0.002	0.010 ± 0.002
*q_max_O2_* (mg O_2_·mg TSS^−1^·h^−1^)	0.102 ± 0.015	0.088 ± 0.017	0.024 ± 0.005	0.022 ± 0.003	0.042 ± 0.010	0.072 ± 0.010*	0.019 ± 0.007	0.021 ± 0.003
*q_max_C2H6_* (mg C_2_H_6_·mg TSS^−1^·h^−1^)	–	0.021 ± 0.002	–	0.008 ± 0.005	–	0.024 ± 0.006	–	0.019 ± 0.004
*μ_max_* (h^−1^)	0.044 ± 0.002	0.022 ± 0.002**	0.020 ± 0.001	0.014 ± 0.002**	0.029 ± 0.002	0.018 ± 0.002**	0.013 ± 0.004	0.012 ± 0.002

^*a*^ Each set comprised varying concentrations of CH_4_ and O_2_: 20% CH_4_ with 30% O_2_ (SET I), 20% CH_4_ with 5.5% O_2_ (SET II), 10% CH_4_ with 30% O_2_ (SET III), and 10% CH_4_ with 5.5% O_2_ (SET IV).

^*b*^ Each parameter represents the maximum specific utilization rate of CH_4_ (*q_max_CH4_*), O_2_ (*q_max_O2_*), and C_2_H_6_ (*q_max_C2H6_*), and maximum specific growth rate (*μ_max_*). Sample codes—Pure CH_4_ and Ethane—denote the absence or presence of 4.5% C_2_H_6_, respectively. Single asterisk (*) and double asterisks (**) are placed next to the kinetic values of the ethane treatments to indicate statistically significant differences from the corresponding Pure CH_4_ controls, with *P*-value < 0.05 and < 0.005, respectively. All experiments were performed in triplicate, with data presented as mean ± SD (File S1).

^*c*^ –, no applicable data.

In line with the decrease in *q_max_CH4_*, the maximum specific growth rates (*μ_max_*) were also considerably attenuated under C_2_H_6_ treatments in SET I, II, and III (Table 1, *P*-value < 0.005). Notably, *μ_max_* values in SET I and III, where CH_4_ was entirely consumed (Fig. 2B and H), also declined significantly upon C_2_H_6_ addition. In type II methanotrophs, formate, a key intermediate in the CH_4_ oxidation pathway, is assimilated into cellular biomass via the serine cycle (53, 54). In contrast, C_2_H_6_ oxidation does not facilitate the formation of assimilable carbon intermediates while simultaneously consuming reducing power, thereby suppressing growth.

Under O_2_-limited SET II and IV, neither CH_4_ nor C_2_H_6_ was fully consumed in any condition due to O_2_ scarcity (Fig. 2D, E, J and K). Unlike SET I and III, where full CH_4_ oxidation occurred under O_2_ sufficiency, the presence of C_2_H_6_ in SET II led to a 37% reduction in net CH_4_ uptake relative to the control (Fig. 2F), indicating that co-substrate effects on CH_4_ consumption are more severe under O_2_ limitation. Comparing C_2_H_6_ treatments between the two O_2_-deficient sets, SET II (20% CH_4_) and SET IV (10% CH_4_), the maximum specific C_2_H_6_ utilization rate (*q_max_C2H6_*) was significantly higher in SET IV (0.019 ± 0.004 mg C_2_H_6_·mg TSS^−1^·h^−1^) than in SET II (0.008 ± 0.005) (Table 1, *P*-value < 0.05). Remarkably, *q_max_C2H6_* in SET IV even exceeded *q_max_CH4_*, highlighting unexpectedly robust C_2_H_6_ oxidation by this obligate methanotroph.

**Fig 3:**
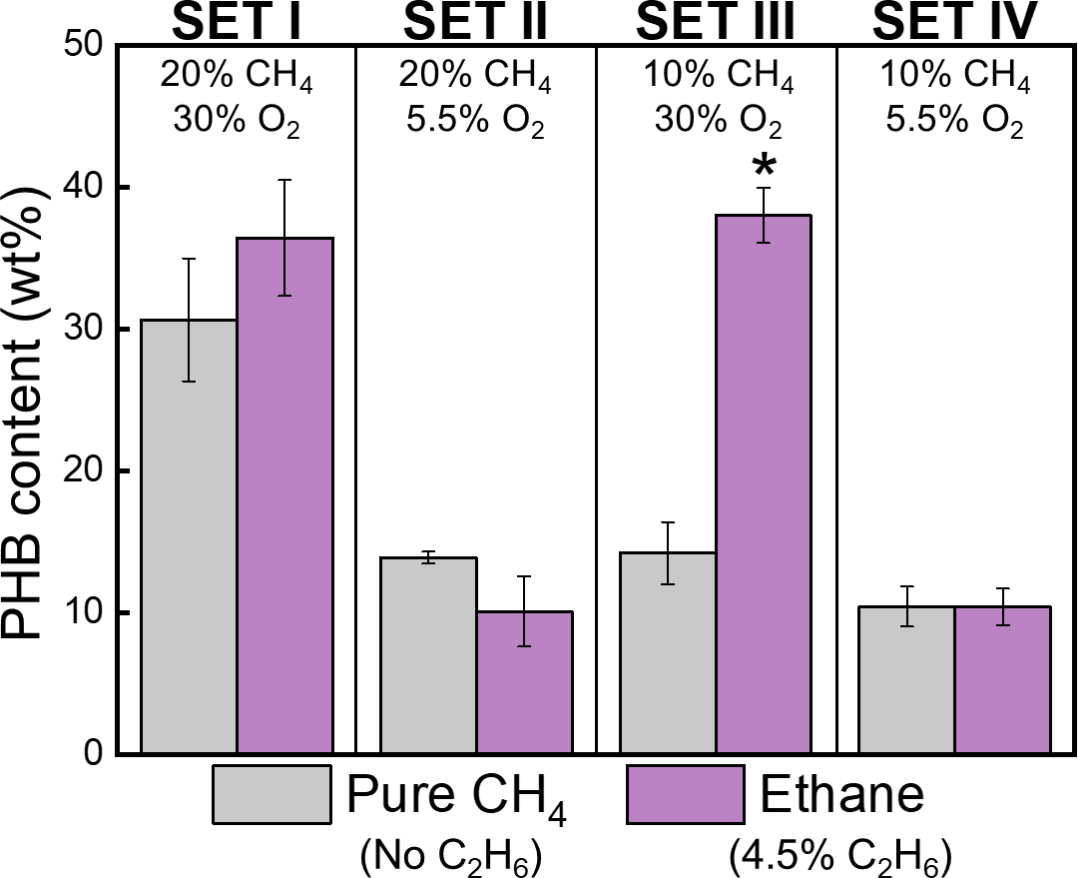
PHB content (wt%) of *M. trichosporium* OB3b cultivated under four distinct sets. SET I and SET III were conducted with 30% O_2_ and 20% CH_4_ (SET I) or 10% CH_4_ (SET III), whereas SET II and SET IV were carried out with 5.5% O_2_ and 20% CH_4_ (SET II) or 10% CH_4_ (SET IV). Each set was further divided into two conditions: a CH_4_-only condition as the control (Pure CH_4_, gray bars) and a 4.5% C_2_H_6_-added condition (Ethane, purple bars). An asterisk (*) indicates a significant difference (*P*-value < 0.005) between the C_2_H_6_ treatments and the pure CH_4_ control. All experiments were performed in triplicate, and data are presented as mean ± SD.

After the nutrient-balanced growth phase, cultures from each set were collected and transferred to nutrient-imbalanced, nitrogen-free media to induce PHB accumulation (Fig. S1). Intriguingly, C_2_H_6_ exposure under O_2_-replete conditions (SET I and III) significantly enhanced PHB synthesis compared with CH_4_-only controls in each set, by 19% and 168%, respectively (Fig. 3, *P*-value < 0.005). In contrast, no such enhancement was observed under O_2_-limited conditions (SET II and IV), where C_2_H_6_ oxidation was incomplete.

Taken together, SET III demonstrated the most pronounced and multifaceted effects of 4.5% C_2_H_6_ addition: a reduction in CH_4_ consumption and growth during nutrient-balanced conditions (despite complete CH_4_ depletion), and a substantial increase in PHB synthesis during nutrient imbalance (Fig. 2 and 3; Table 1). To further explore these effects, additional experiments with varying C_2_H_6_ concentrations were conducted under SET III (10% CH_4_ with 30% O_2_).

### Effects of varying C_2_H_6_ concentrations under SET III

Under SET III conditions, with deficient CH_4_ (10%, vol/vol) and excess O_2_ (30%, vol/vol), we investigated five C_2_H_6_ concentrations—0%, 5%, 10%, 20%, and 50%—designated as E0, E5, E10, E20, and E50, respectively. Additionally, to verify the obligate methanotrophy of *M. trichosporium* OB3b, an extra condition termed ObliE20 was included, where 20% C_2_H_6_ and 30% O_2_ were supplied in the absence of CH_4_ (File S2).

**Fig 4:**
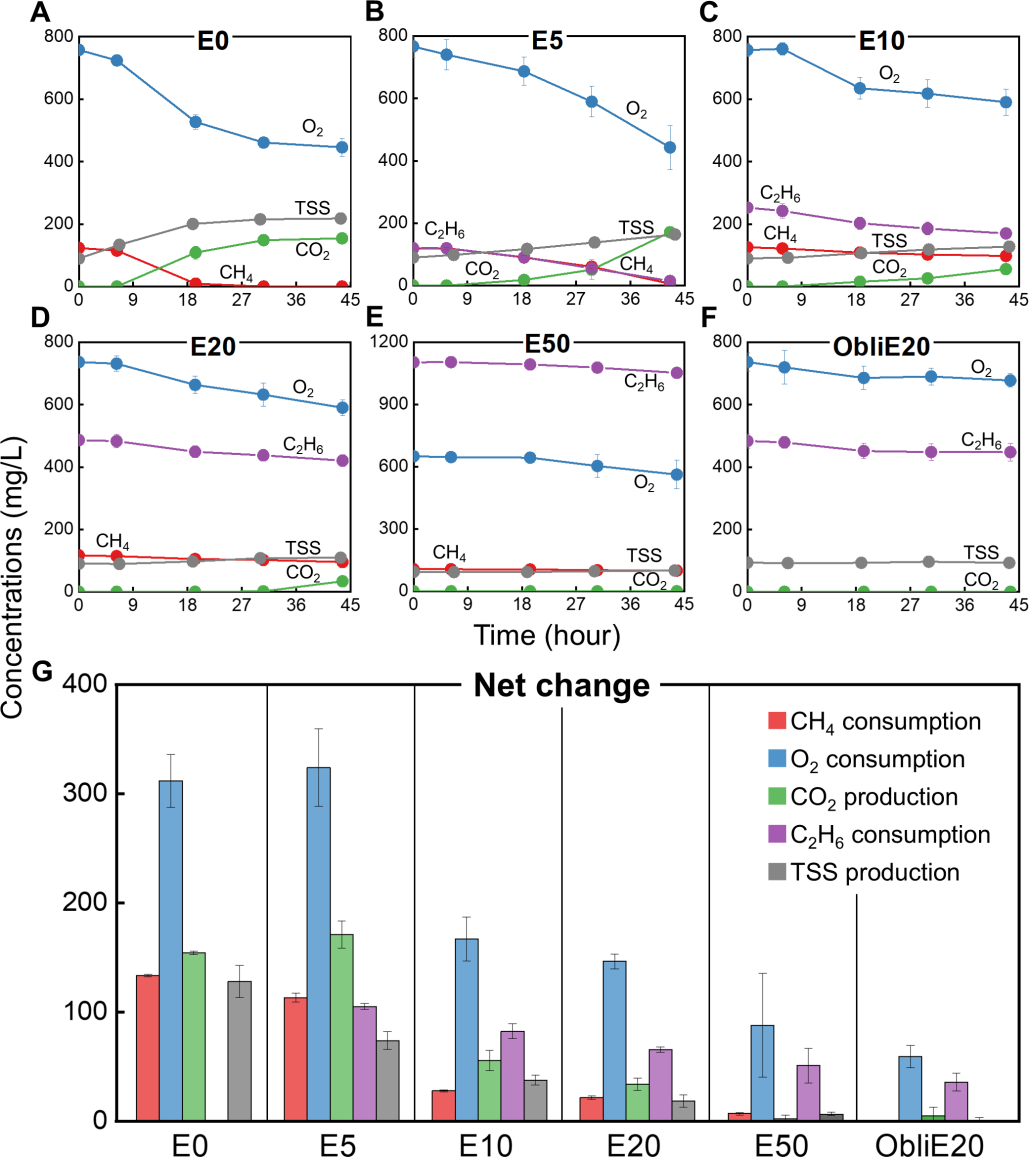
Activity of *M. trichosporium* OB3b cultures under SET III conditions (10% CH_4_ and 30% O_2_, vol/vol) with varying concentrations of C_2_H_6_. Shown are the consumption of CH_4_ (red), O_2_ (blue), and C_2_H_6_ (purple), and the production of CO_2_ (green) and TSS (gray). The five distinct conditions—E0 (**A**), E5 (**B**), E10 (**C**), E20 (**D**), and E50 (**E**)—correspond to 0%, 5%, 10%, 20%, and 50% C_2_H_6_ (vol/vol), respectively. An additional condition, ObliE20 (**F**), contained 20% C_2_H_6_ without CH_4_. Net changes in CH_4_, O_2_, and C_2_H_6_ (consumed), and CO_2_ and TSS (produced), during the 45 h cultivation are depicted in (**G**). Y-axes represent nominal concentrations (mg/L), calculated as the total mass per bottle divided by the liquid-phase volume (0.05 L). All experiments were performed in triplicate, and the results are shown as mean ± SD (File S2).

In ObliE20 (Fig. 4F and G), no increase in biomass was observed, confirming that *M. trichosporium* OB3b is incapable of using C_2_H_6_ as a growth substrate and thus retains obligate methanotrophic characteristics. Nevertheless, a small amount of C_2_H_6_ was consumed in ObliE20 (35.9 ± 8.20 mg/L), which was significantly lower than in E20 (65.7 ± 2.48 mg/L), where the same C_2_H_6_ concentration (20%) was provided (*P*-value < 0.005). This resonates with the aforementioned dependence of C_2_H_6_ oxidation on CH_4_ availability, which serves as an indispensable source of reducing equivalents, energy, and overall metabolic activity.

Interestingly, complete CH_4_ consumption occurred only in E0 and E5 (Fig. 4A and B). This finding differs from previous SET I and III results, in which CH_4_ was fully consumed even in the presence of 4.5% C_2_H_6_ (Fig. 2B and H). In the current experiment, however, when C_2_H_6_ concentrations exceeded 5% (i.e., E10–ObliE20), not only *q_max_CH4_* but also total CH_4_ uptake declined sharply (Table 2; Fig. S3), despite constant O_2_ availability (30%) as in SET I and III. These findings highlight a threshold effect of C_2_H_6_ concentration on CH_4_ oxidation, which was not evident at lower C_2_H_6_ levels (< 5%) in previous experiments.

**TABLE 2:** Kinetic parameters of *M. trichosporium* OB3b supplied with 0–50% C_2_H_6_ and 30% O_2_, with or without 10% CH_4_ (vol/vol) during the nutrient-balanced growth phase^*a*^^,^^*b*^

Sample codes (% C_2_H_6_)	E0 (0%)	E5 (5%)	E10 (10%)	E20 (20%)	E50 (50%)	ObliE20 (20%)
*q_max_CH4_* (mg CH_4_·mg TSS^−1^·h^−1^)	0.038 ± 0.001	0.021 ± 0.001**	0.006 ± 0.0001**	0.005 ± 0.001**	0.002 ± 0.0003**	–
*q_max_O2_* (mg O_2_·mg TSS^−1^·h^−1^)	0.079 ± 0.003	0.063 ± 0.006*	0.030 ± 0.003**	0.031 ± 0.003**	0.021 ± 0.010**	0.017 ± 0.003**
*q_max_C2H6_* (mg C_2_H_6_·mg TSS^−1^·h^−1^)	–	0.019 ± 0.001	0.018 ± 0.002	0.014 ± 0.001	0.011 ± 0.004	0.009 ± 0.001
*μ_max_* (h^−1^)	0.041 ± 0.003	0.014 ± 0.001**	0.009 ± 0.001**	0.005 ± 0.002**	0.002 ± 0.001**	–
*T_y_* (mg C_2_H_6_·mg CH_4_^−1^)	–	0.93 ± 0.01	2.96 ± 0.19	3.07 ± 0.12	7.27 ± 1.02	–
Inhibition index (*i*)	–	0.663	0.791	0.876	0.959	–

^*a*^ Each parameter stands for the maximum specific utilization rates for CH_4_ (*q_max_CH4_*), O_2_ (*q_max_O2_*), and C_2_H_6_ (*q_max_C2H6_*); the maximum specific growth rate (*μ_max_*); transformation yield (*T_y_*); and inhibition index (*i*). The inhibition index (*i*) is calculated as [(*μ_max_control_ - μ_max_sample_*)·*μ_max_control_*^−1^] (55). Sample codes—E0, E5, E10, E20, E50—denote different C_2_H_6_ concentrations of 0%, 5%, 10%, 20%, and 50% (vol/vol), respectively. Although E5, E10, E20, and E50 involved 10% CH_4_ with specific concentrations of C_2_H_6_, ObliE20 consisted of 20% C_2_H_6_ without CH_4_. Single asterisk (*) and double asterisks (**) next to the kinetic values of the E5–E50 treatments indicate statistically significant differences compared from the CH_4_-only E0 control, with *P*-value < 0.05 and < 0.001, respectively. All conditions were supplied with 30% O_2_. Experiments were carried out in triplicate, and data are presented as mean ± SD (File S2).

^*b*^ –, no applicable data.

As C_2_H_6_ concentrations increased, the amount of C_2_H_6_ consumed also progressively declined (Fig. 4G; Fig. S3), with complete C_2_H_6_ oxidation achieved only in E5 (Fig. 4B). Notably, the reduction in CH_4_ uptake with rising C_2_H_6_ levels was substantially steeper than the corresponding decrease in C_2_H_6_ consumption (Fig. S3). Specifically, total C_2_H_6_ consumption decreased by 21.6% in E10 relative to E5, whereas total CH_4_ consumption dropped sharply by 75.4% under the same conditions. Regarding specific utilization rates, as C_2_H_6_ concentrations increased from 0% to 50%, *q_max_C2H6_* exhibited a linear decline (R^2^ = 0.904), whereas *q_max_CH4_* followed an exponential decay model (R^2^ = 0.971), with *q_max_C2H6_* exceeding *q_max_CH4_* at C_2_H_6_ concentrations above 10% (Fig. S3; Table 2). This pattern suggests unexpectedly robust and sustained C_2_H_6_ oxidation, further supported by the transformation yield (*T_y_*)—defined as the ratio of non-growth substrate (C_2_H_6_) consumed to growth substrate (CH_4_) consumed—which quantifies the extent of co-metabolism facilitated by CH_4_ oxidation (44, 45, 56). *T_y_* values increased proportionally with C_2_H_6_ concentration, implying that CH_4_ was increasingly diverted toward supporting C_2_H_6_ oxidation at higher concentrations.

Elevated C_2_H_6_ concentrations also suppressed cell growth. Even in E5, where CH_4_ was entirely depleted as in E0, biomass production was lower than in E0 (Fig. 4G; Fig. S2). This observation aligns with SET I and III results (Fig. 2C and I), where 4.5% C_2_H_6_ reduced biomass assimilation compared with the pure CH_4_ controls, despite complete CH_4_ consumption. Mirroring the trends in *q_max_CH4_*, *μ_max_* decreased, and the inhibition index (*i*) increased exponentially with rising C_2_H_6_ concentrations (R^2^ = 0.969 and 0.992, respectively). These findings indicate that high C_2_H_6_ levels impose strong inhibitory effects on the kinetics of both CH_4_ consumption and biomass growth (Table 2; Fig. S3).

Although C_2_H_6_ consistently had detrimental effects on both CH_4_ consumption and biomass production during the nutrient-balanced growth phase, a positive correlation between C_2_H_6_ concentration and PHB content was observed during the nutrient-imbalanced phase (Table 3). A PHB content of 6.57 wt% was detected in ObliE20, although this was substantially lower than those observed in C_2_H_6_-supplemented conditions that included CH_4_.

**TABLE 3:** PHB content (wt%) of *M. trichosporium* OB3b supplied with 30% O_2_ along with 0–50% C_2_H_6_, with or without 10% CH_4_ (vol/vol)^*a*^

Sample codes (% C_2_H_6_)	E0 (0%)	E5 (5%)	E10 (10%)	E20 (20%)	E50 (50%)	ObliE20 (20%)
PHB content (wt%)	9.43 ± 1.74	30.8 ± 1.09**	30.4 ± 4.98**	37.1 ± 4.89**	48.4 ± 15.2*	6.57 ± 5.47

^*a*^ The sample codes—E0, E5, E10, E20, and E50—refer to cultures supplied with C_2_H_6_ concentrations of 0%, 5%, 10%, 20%, and 50% (vol/vol), respectively. ObliE20 involved 20% C_2_H_6_ and 30% O_2_ in the absence of 10% CH_4_. Single asterisk (*) and double asterisks (**) denote significant differences (*P*-value < 0.05 and < 0.005, respectively) between C_2_H_6_-treated conditions and the control (E0). Each experiment was carried out in triplicate, and data are presented as mean ± SD.

### Testing three hypotheses

Across all preceding experiments, the addition of C_2_H_6_ consistently impaired CH_4_ consumption (as evidenced by reduced total CH_4_ consumption and *q_max_CH4_*), restrained cell growth (lower total biomass increase and *μ_max_*), and promoted PHB synthesis. To elucidate the mechanisms underlying these trends, we proposed three hypotheses:

#### MMO-mediated C_2_H_6_ oxidation depletes reducing power, hindering CH_4_ oxidation

Both CH_4_ and C_2_H_6_ are oxidized via MMO, which necessitates two units of reducing equivalents for substrate activation (32). The observed preference for C_2_H_6_ oxidation—evidenced by the exponential decrease in CH_4_ utilization compared with the linear decline in C_2_H_6_ uptake and the increase in *T_y_* values as C_2_H_6_ concentrations increased (Fig. S3; Table 2)—suggests that excess C_2_H_6_ may compete for reducing power, thus restricting CH_4_ metabolism (45, 56, 57). To test this, we supplemented the cultures with methanol or sodium formate as external sources of reducing equivalents (47, 58).

During a 45 h growth phase, 8.85 mM sodium formate, corresponding to the theoretical formate yield from the complete oxidation of 10% CH_4_, was provided in E20 and ObliE20, designated FE20 and FobliE20, respectively (Fig. S4). In both FE20 and FobliE20, formate addition neither significantly affected CH_4_, O_2_, or C_2_H_6_ consumption nor biomass production compared with their respective controls, E20 and ObliE20 (*P*-value > 0.1). However, only the net CO_2_ production increased appreciably in both FE20 and FobliE20 compared with E20 and ObliE20, confirming that the supplied formate was indeed oxidized (Fig. S4*, P-*value < 0.001). Although exogenous formate is known to supply reducing power to methanotrophs (12, 29, 45, 47), it failed to alleviate the C_2_H_6_-induced CH_4_ uptake impediment in our study (Fig. S4). PHB content was also unaffected by formate addition (FE20 vs. E20 and FobliE20 vs. ObliE20) (Table 4, *P*-value > 0.5).

**TABLE 4:** PHB content (wt%) of *M. trichosporium* OB3b cultivated with 30% O_2_ and C_2_H_6_ or sodium formate, with or without 10% CH_4_ (vol/vol)^*a*^

Sample codes	E0 (10% CH_4_, 0% C_2_H_6_)	E20 (10% CH_4_, 20% C_2_H_6_)	FE20 (10% CH_4_, 20% C_2_H_6_, formate)	FobliE20 (0% CH_4_, 20% C_2_H_6_, formate)	ObliE20 (0% CH_4_, 20% C_2_H_6_)
PHB content (wt%)	9.92 ± 1.32	34.4 ± 3.41*	36.6 ± 2.86*	7.60 ± 3.75	5.27 ± 3.63

^*a*^ Except for FobliE20 and ObliE20, all other conditions (E0, AC20, E20, and FE20) included 10% CH_4_. C_2_H_6_ was present at 20% in E20, FE20, FobliE20, and ObliE20. Sodium formate (8.85 mM) was provided in FE20 and FobliE20. An asterisk (*) denotes a statistically significant difference (*P-value* < 0.001) compared with E0. All experiments were performed in triplicate, and results are presented as mean ± SD.

**Fig 5:**
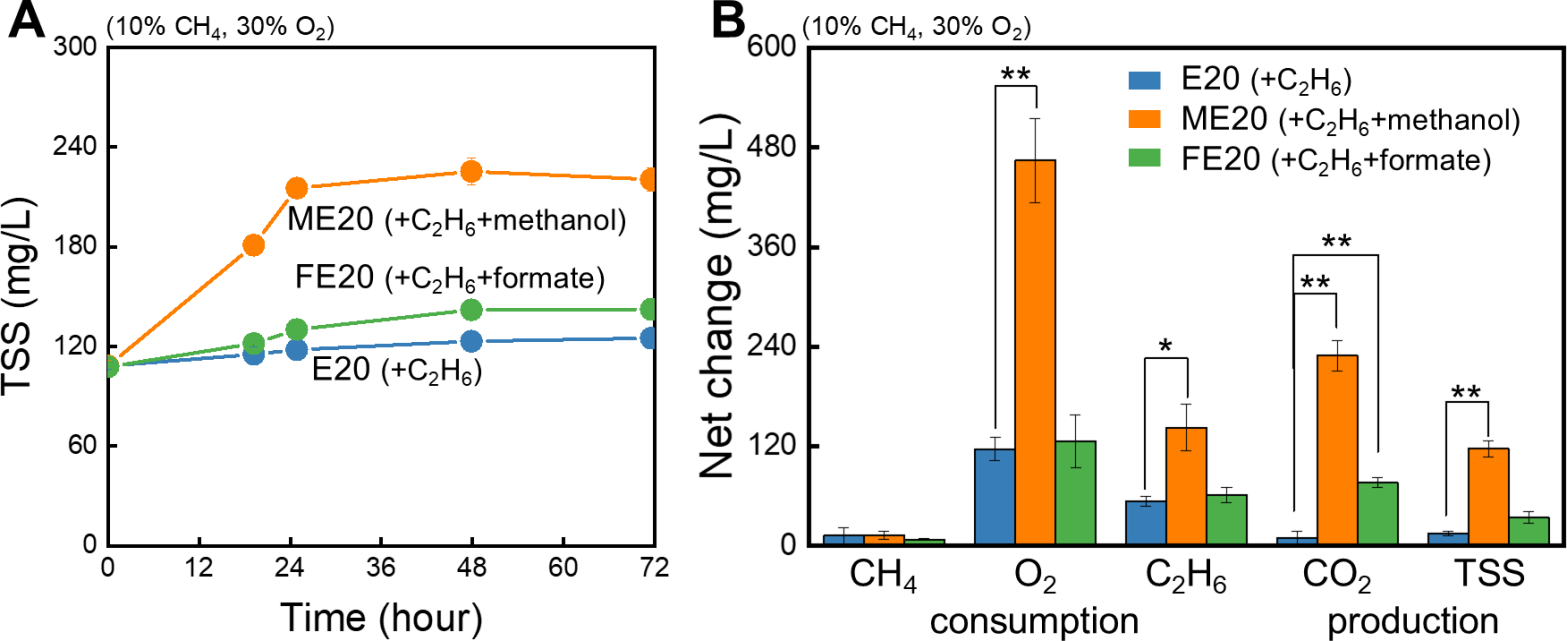
TSS increase (**A**) and net changes in consumption (CH_4_, O_2_, and C_2_H_6_) and production (CO_2_ and TSS) (**B**) by *M. trichosporium* OB3b during the 72 h growth phase. Blue, orange, and green data represent the E20, ME20, and FE20 conditions, respectively. E20 was supplied with 20% C_2_H_6_ under SET III condition (10% CH_4_, 30% O_2_, vol/vol). In ME20 and FE20, 8.85 mM methanol or formate, respectively, was supplemented to the E20 condition. Single (*) and double asterisks (**) indicate significant differences (*P*-value < 0.05 and < 0.001, respectively). Concentrations shown on the Y-axes are nominal, obtained by dividing the total amount of each component by the liquid-phase volume (0.05 L). All experiments were carried out in triplicate, and data are reported as mean ± SD.

In contrast, ME20—an E20 condition supplemented with 8.85 mM methanol—significantly enhanced cell growth (*µ_max_* = 0.027 ± 0.001 h^−1^), with methanol serving as an additional assimilable carbon source (Fig. 5A*, P-*value < 0.001). Interestingly, although methanol did not restore CH_4_ oxidation—which remained low, as in E20 and FE20 (*P*-value = 0.6)—it substantially promoted C_2_H_6_ consumption (Fig. 5B*, P-*value = 0.006). This improved C_2_H_6_ uptake in ME20 was consistent with significantly higher CO_2_ production and O_2_ consumption compared with E20 and FE20 (*P*-value < 0.001). Methanol was almost completely consumed within 48 h across all replicates (Table S1), suggesting that the reducing equivalents from methanol oxidation were utilized to support MMO-mediated C_2_H_6_ oxidation in ME20.

The contrasting effects of formate and methanol on C_2_H_6_ consumption in FE20 and ME20 likely arise from differences in their electron yields. Formate oxidation releases only 2 moles of electrons per mole (51), whereas methanol yields 6 (equation 3, [51]), indicating that formate addition is insufficient to induce a substantial increase in C_2_H_6_ oxidation. More intriguingly, the reducing equivalents supplied by methanol appear to be preferentially funneled into C_2_H_6_ uptake rather than CH_4_ metabolism, despite C_2_H_6_ being a non-growth substrate. This puzzling preference for reducing power allocation toward C_2_H_6_ oxidation aligns with preceding findings, in which increasing C_2_H_6_ concentrations led to a sharp decline in CH_4_ consumption, whereas C_2_H_6_ consumption decreased more progressively, resulting in *q_max_C2H6_* exceeding *q_max_CH4_* at concentrations above 10% (Fig. S3; Table 2; File S2).

#### Acetate derived from C_2_H_6_ oxidation may inhibit microbial growth or promote PHB accumulation

C_2_H_6_ is sequentially oxidized to ethanol (via MMO), acetaldehyde (via alcohol dehydrogenase), and ultimately to acetate (via acetaldehyde dehydrogenase) (28, 32, 59). Acetate formation was confirmed in the medium of C_2_H_6_-supplied conditions after the PHB production phase (Table S2). Previous studies have reported that acetate accumulation can inhibit microbial growth (24, 27, 55, 60), while also serving as a precursor to PHB through conversion to acetyl-CoA, the entry point of the PHB synthesis pathway (32, 61, 62). To test this hypothesis, a medium amended with 17.7 mM sodium acetate, corresponding to the theoretical acetate yield from the complete oxidation of 20% C_2_H_6_, was used in the AC20 condition.

**Fig 6:**
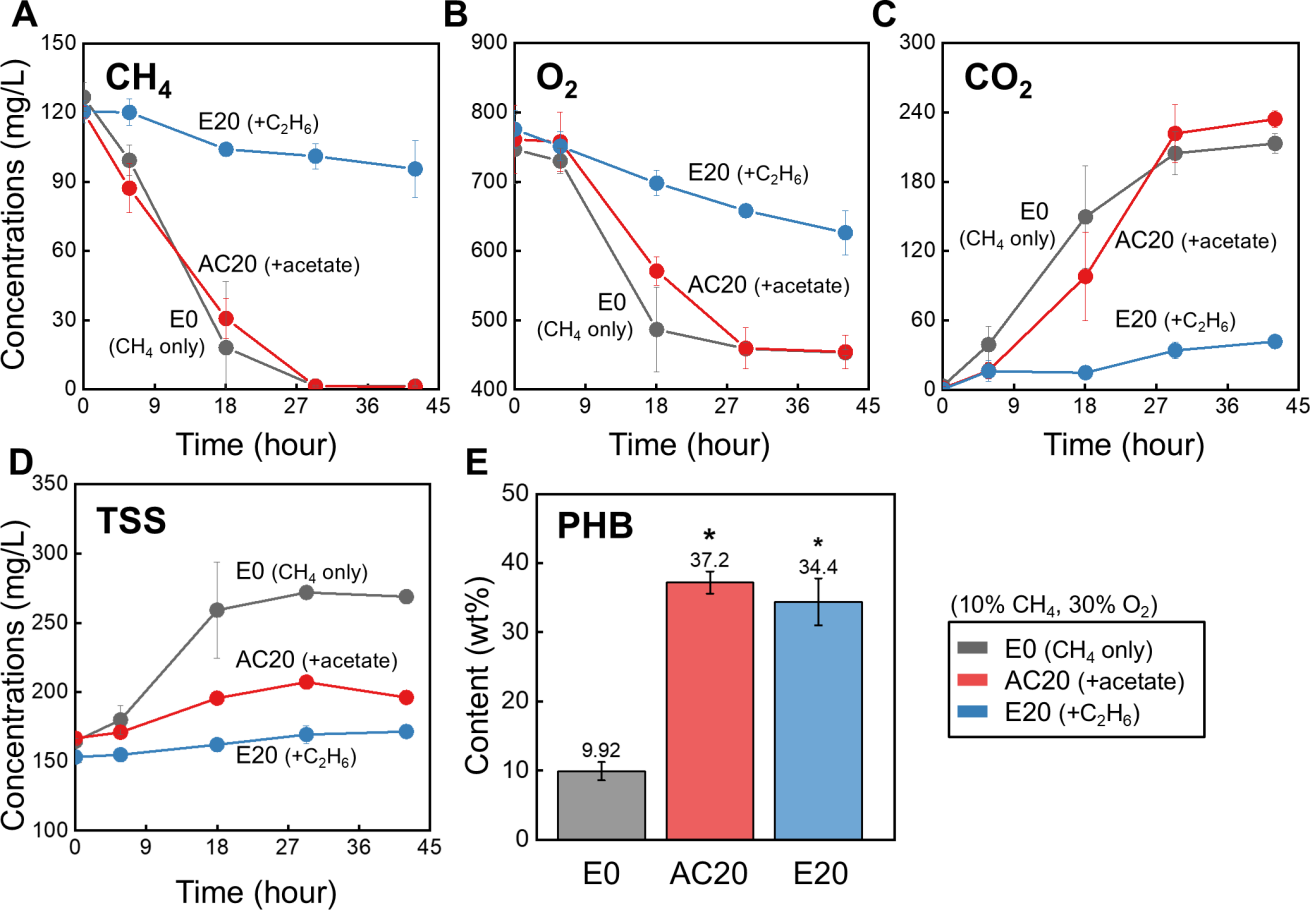
Activity of *M. trichosporium* OB3b cultures under SET III conditions (10% CH_4_ and 30% O_2_, vol/vol) with 20% C_2_H_6_ (E20, blue) or 17.7 mM sodium acetate (AC20, red). The control condition, E0 (gray), included neither C_2_H_6_ nor acetate. Panels show the consumption of CH_4_ (**A**) and O_2_ (**B**), production of CO_2_ (**C**) and TSS (**D**) during the 45 h growth phase, and PHB content (**E**) measured after the PHB accumulation phase. Y-axes in panels (A–D) show nominal aqueous-phase concentrations, normalized to a liquid volume of 0.05 L. Single asterisk (*) indicates a significant difference (*P*-value < 0.001). All experiments were conducted in triplicate, and the results are shown as mean ± SD.

Complete consumption of 10% CH_4_ occurred not only in E0 but also in AC20 (Fig. 6A), indicating that acetate does not inhibit MMO activity. The *q_max_CH4_* values of E0 (0.032 ± 0.004 mg CH_4_·mg TSS^−1^·h^−1^) and AC20 (0.027 ± 0.002) were statistically comparable (*P*-value > 0.05). However, biomass production in AC20 was significantly lower than in E0 (Fig. 6D*, P-*value < 0.001), suggesting that C_2_H_6_-derived acetate impairs growth despite not interfering directly with CH_4_ oxidation. As acetate concentration increased (0–26.5 mM), biomass decreased proportionally (Fig. S5). Moreover, biomass accumulation in E20 was even lower than in AC20 (*P*-value < 0.01), indicating that growth inhibition in E20 was not solely attributable to acetate toxicity, but rather to a combined effect of acetate-derived toxicity and the reduced CH_4_ consumption (i.e., reduced growth substrate uptake), compounding the growth suppression observed in all C_2_H_6_ treatments.

Although this study, centered on C_2_H_6_, proposes that growth inhibition by C_2_H_6_ may primarily stem from C_2_H_6_-derived acetate, several mechanisms for acetate toxicity have been suggested in prior acetate-focused studies (24, 27, 55, 63–69). One classical hypothesis posits that undissociated acetic acid diffuses and dissociates within the cell, depleting the proton motive force and causing energetically costly proton efflux to maintain membrane potential (24, 27, 65). Another theory suggests that acetate accumulation disrupts intracellular anion balance, triggering the secretion of alternative anions to balance osmotic pressure (63, 64). A further hypothesis postulates that elevated acetyl-phosphate levels by excess acetate influx may act as a signaling molecule, modulating gene expression and metabolic processes, thereby ultimately impairing growth (70, 71). Although a full mechanistic dissection is beyond the scope of this study, our results highlight how metabolic intermediates—such as acetate—generated under co-metabolic conditions can perturb methanotrophic physiology in complex and unpredictable ways.

Despite its growth-inhibitory effect, both C_2_H_6_ (E20) and acetate (AC20) appreciably enhanced PHB synthesis compared with the CH_4_-only condition (E0) (Fig. 6E*, P-*value < 0.001). This increase in PHB accumulation by C_2_H_6_ or acetate was consistent across a range of C_2_H_6_ (0%–50%) and acetate (0–26.5 mM) concentrations (Table 3; Fig. S5). PHB production in type II methanotrophs begins with acetyl-CoA, followed by a series of enzymatic steps (29, 32). Therefore, increasing the intracellular pool of acetyl-CoA, the pivotal building block for PHB synthesis, facilitates PHB formation (61, 72). Two known microbial pathways convert acetate to acetyl-CoA: (i) the reversible acetate kinase (ACK)-phosphate acetyltransferase (PTA) pathway and (ii) the irreversible acetyl-CoA synthetase (ACS) pathway (14, 73, 74). In the ACK-PTA pathway, acetate is first phosphorylated by ACK to form acetyl phosphate, which is then converted to acetyl-CoA by PTA (72, 73).

To investigate the likely pathway for acetyl-CoA synthesis from acetate in *M. trichosporium* OB3b, we examined its genome and protein databases using the NCBI (National Center for Biotechnology Information) and UniProt resources. Although neither ACS nor its encoding gene could be identified, ACK (accession number: ATQ69175) and PTA (ATQ69176) were detected, suggesting that *M. trichosporium* OB3b likely uses the ACK-PTA pathway for acetate assimilation. In the absence of C_2_H_6_ or acetate assimilation, acetyl-CoA must be generated solely via the serine cycle (75), explaining the comparatively lower PHB production in the pure CH_4_ controls compared with C_2_H_6_-/acetate-supplemented cultures.

Previous studies using acetate-fed type II obligate methanotrophs have confirmed dynamic acetate incorporation into PHB, as shown by ^13^C NMR spectroscopy (76). Another study revealed that the oxidation of C1 compounds and primary alcohols supported the assimilation of ^14^C-labeled acetate by *M. trichosporium* Pa, implying the feasibility of acetate assimilation metabolism (77, 78). Additionally, our previous study also demonstrated that *Methylocystis parvus* OBBP, a type II obligate methanotroph, incorporated ^13^C-labeled C_2_H_6_ into PHB in the absence of CH_4_, although no growth was observed during the nutrient-balanced growth phase (32).

In summary, C_2_H_6_-derived acetate exerted opposing effects depending on the nutrient regime: growth suppression under nutrient-balanced conditions and PHB promotion under nutrient imbalance. These results suggest that acetate metabolism and carbon storage in type II obligate methanotrophs are highly responsive to changes in both nutrient availability and substrate composition.

#### C_2_H_6_ may modulate MMO gene expression, influencing CH_4_ oxidation during co-metabolism

**Fig 7:**
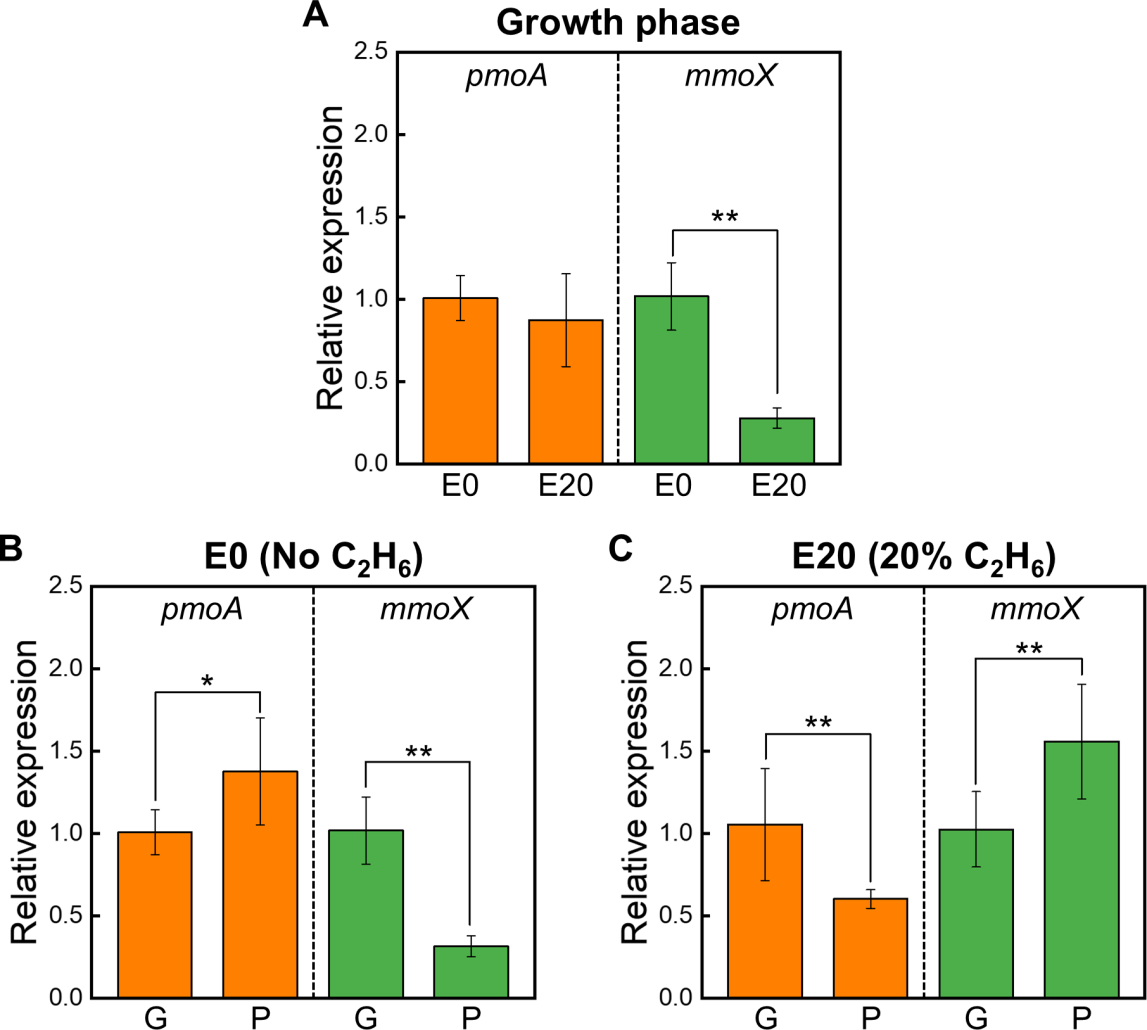
RT-qPCR analysis of the relative expression levels of *pmoA* (orange bars) and *mmoX* (green bars) in *M. trichosporium* OB3b cultivated under either E0 (0% C_2_H_6_) or E20 (20% C_2_H_6_) during the nutrient-balanced cell growth (G) and nutrient-imbalanced PHB accumulation (P) phases. The upper panel (A) shows gene expression during the G phase under E0 and E20. The lower panels express phase-dependent expression changes between the G and P phases under E0 (B) and E20 (C). Relative expression of the target genes was quantified using the *2*^*−ΔΔCt*^ method, with 16S rRNA as the reference gene for normalization. Single (*) and double asterisks (**) denote significant differences (*P-*value < 0.01 and < 0.001, respectively). All data are presented as mean ± SD (File S3).

*M. trichosporium* OB3b expresses both soluble MMO (sMMO) and particulate methane monooxygenase (pMMO), both of which are capable of oxidizing CH_4_ and C_2_H_6_ (42). It is therefore reasonable to hypothesize that C_2_H_6_ exposure could directly influence the expression of *mmoX* and *pmoA*, the genes encoding sMMO and pMMO, respectively, potentially altering the efficiency of CH_4_ oxidation. To test this hypothesis, we conducted quantitative real-time reverse transcription PCR (RT-qPCR) to assess the relative expression of *pmoA* and *mmoX* in the presence or absence of C_2_H_6_ during the growth phase and PHB accumulation phase (Fig. 7; Fig. S6; File S3).

During the growth phase, *mmoX* expression was explicitly downregulated in E20 (*P*-value < 0.001), whereas *pmoA* expression remained unchanged (*P*-value > 0.2) (Fig. 7A). Despite the repression of *mmoX* in E20, the unchanged expression of *pmoA* (Fig. 7A), combined with the inherently lower *mmoX* expression relative to *pmoA* under Cu-sufficient conditions (79), suggests that the reduction in CH_4_ uptake by C_2_H_6_ addition is more likely explained by other factors, such as reoriented distribution of reducing equivalents (Fig. 5) or compromised cell viability by acetate accumulation (Fig. 6; Fig. S5), rather than direct transcriptional inhibition of MMO genes.

The unexpectedly stable *pmoA* transcript levels in E20, despite a marked reduction in CH_4_ consumption compared with E0 (Fig. 4D and G), challenge the common assumption that mRNA abundance assessed by RT-qPCR reliably reflects enzymatic activity or actual metabolic function (80, 81). Although *pmoA* is widely harnessed as a functional marker for methanotrophic activity due to the near-universal presence of pMMO among methanotrophs (14, 43, 80), our findings suggest a potential disconnect between MMO gene transcript abundance and actual CH_4_ oxidation capacity, particularly under co-substrate conditions.

In the CH_4_-only condition (E0), *pmoA* transcription increased when transitioning from the growth phase to the PHB production phase (Fig. 7B*, P-*value < 0.01), whereas *mmoX* was significantly downregulated (*P*-value < 0.001). In contrast, under C_2_H_6_-amended conditions (E20), this trend reversed in the PHB phase: *pmoA* was downregulated, whereas *mmoX* was upregulated relative to the prior growth phase (Fig. 7C*, P-*value < 0.001). These results indicate that C_2_H_6_ exposure distinctly modulates MMO gene expression depending on the metabolic phase, specifically in response to nutrient balance or stress. This phase-dependent transcriptional response highlights an adaptive modulation of methanotrophic metabolism under C_2_H_6_ influence, suggesting that MMO gene expression in *M. trichosporium* OB3b is not only responsive to nutrient status but also sensitive to co-substrate exposure.

### Conclusions

This study elucidates the intricate interplay between methanotrophic activity and the co-metabolism of the non-growth substrate C_2_H_6_ in *M. trichosporium* OB3b, a type II obligate methanotroph. Although C_2_H_6_ does not support biomass growth, its oxidation, when co-supplied with CH_4_, significantly perturbs methanotrophic metabolism by constraining CH_4_ consumption and growth while enhancing PHB synthesis. Two main factors drive these effects: competition for reducing power, favoring C_2_H_6_ oxidation over CH_4_ uptake, and the dual role of C_2_H_6_-derived acetate on carbon assimilation, acting as both an inhibitor of growth and a precursor for PHB biosynthesis. Notably, the relative expression levels of MMO genes did not mirror the observed decrease in CH_4_ consumption, highlighting a potential discrepancy between gene expression and enzymatic activity under co-metabolic conditions.

Although our approach provided critical insights into the hypothesized mechanisms, it did not encompass the entire spectrum of metabolic adaptations, such as unexpected shifts in central carbon metabolism or cellular stress responses. To comprehensively resolve system-wide physiological changes under C_2_H_6_ exposure, future studies should include transcriptomic or metabolomic analysis, which is essential to scrutinize metabolic fluxes and regulatory networks at a global scale. Additionally, comparative investigations involving both obligate and facultative methanotrophs are needed to clarify conserved versus divergent C_2_H_6_ co-metabolic strategies across methanotrophic lineages.

These findings shed light on the metabolic variability of methanotrophs in heterogeneous substrate environments, where non-growth substrates interact with CH_4_ metabolism in complex ways. Furthermore, this study emphasizes the importance of accounting for microbial metabolites and by-products, such as methanol and acetate, when evaluating methanotrophic activity—particularly in ecosystems with fluctuating nutrient availabilities (e.g., CH_4_, O_2_, and nitrogen sources) or the presence of co-metabolic substrates (e.g., C_2_H_6_). These insights provide a foundation for the optimization of methanotroph-based biotechnological processes, such as biopolymer production and CH_4_ mitigation, under diverse environmental conditions.

## MATERIALS AND METHODS

### Materials and reagents

Unless otherwise stated, all chemicals and reagents were purchased from Sigma-Aldrich (St. Louis, MO, USA) and were of analytical grade or bioreagent grade with higher purity. All gases, including CH_4_, O_2_, and C_2_H_6_ (purity ≥ 99.9%; Jeil Gas Industry Co., Ltd., Incheon, Republic of Korea), were injected through a 0.20 µm filter (ADVANTEC Toyo Roshi Kaisha, Ltd., Tokyo, Japan) equipped with a gastight syringe.

### Strain and culture conditions

One of the representative type II methanotrophs, *M. trichosporium* OB3b, was cultivated in batch cultures under strict sterile conditions. The strain was obtained from the Korean Collection for Type Cultures (KCTC, Jeongeup, Republic of Korea). This type II methanotroph was selected based on its well-documented metabolomic and stoichiometric information (82, 83). This strain has been reported as an obligate methanotroph (84) that can only grow on C1 substrate (i.e., CH_4_ or methanol) but has never been cultured on the mixture of CH_4_ and C_2_H_6_ under CH_4_ and/or O_2_ replete/deplete conditions.

Pure cultures of *M. trichosporium* OB3b were cultivated in nitrate mineral salt medium (NMS) in 160 mL serum bottles. The NMS medium contained the following chemicals (per 1 L): 1.0 g of MgSO_4_∙7H_2_O, 1.0 g of KNO_3_, 0.2 g of CaCl_2_∙2H_2_O, 0.5 mL of a 0.1% (wt/vol) Na_2_MoO_4_∙2H_2_O solution, 0.1 mL of a 3.8% (wt/vol) Fe(III)-EDTA solution, and 1 mL of a trace stock solution. The trace stock solution contained (per 1 L): 500 mg of FeSO_4_∙7H_2_O, 400 mg of ZnSO_4_∙7H_2_O, 20 mg of MnCl_2_∙7H_2_O, 50 mg of CoCl_2_∙6H_2_O, 10 mg of NiCl_2_∙6H_2_O, 15 mg of H_3_BO_3_, and 250 mg of EDTA.

Serum bottles were filled with 43 mL of NMS medium, capped with butyl-rubber stoppers, and crimp-sealed. After autoclaving (121°C, 40 min), 0.5 mL of a phosphate buffer solution (containing 26 g of KH_2_PO_4_ and 33 g of Na_2_HPO_4_ per 1 L) adjusted to pH 6.8, 0.5 mL of 1-fold concentrated filter-sterilized vitamin stock (containing 2.0 mg of biotin, 2.0 mg of folic acid, 5.0 mg of thiamine HCl, 5.0 mg of Ca pantothenate, 0.1 mg of vitamin B12, 5.0 mg of riboflavin, and 5.0 mg nicotinamide per 1 L), and 1 mL of a 0.5 mM CuCl_2_∙2H_2_O solution were added to each serum bottle containing 43 mL medium. All bottles consisted of 50 mL of the total liquid phase (inoculum 10%, vol/vol) and 110 mL of headspace. The headspace was filled with methane (CH_4_), oxygen (O_2_), argon (Ar), and ethane (C_2_H_6_) at specific ratios, as detailed in the following sections *5.4*, *5.5*, and *5.6*. Serum bottles were horizontally agitated in a shaking incubator at 150 rpm, 30°C.

To promote polyhydroxybutyrate (PHB) accumulation in cells, the traditional two-phase cultivation method, also known as the feast-famine strategy (phase 1: nutrient-balanced growth phase; phase 2: PHB accumulation phase), was applied (Fig. S1). Both phases were carried out in the same NMS medium as previously described, except for the nitrogen source (nitrate, in this study), which was excluded in the PHB production phase. After the nutrient-balanced growth phase, all cell cultures were harvested and centrifuged at 3,500 rpm for 20 min. Leaving only 5 mL, the supernatant was discarded, and the vortexed pellets were resuspended in a fresh nitrogen-depleted NMS medium.

### Culture purity test

To verify the culture purity of *M. trichosporium* OB3b, genomic DNA of cell cultures was extracted using the DNeasy PowerMax Soil Kit (Qiagen, Hilden, Germany) following the manufacturer’s protocol. Extracted DNA samples were sent to Macrogen Inc. (Daejeon, Republic of Korea), amplified through polymerase chain reaction (PCR), and sequenced. In PCR, bacterial 16S rRNA genes were amplified using the bacterial universal primers, 27F (5′-AGAGTTTGATCMTGGCTCAG-3′) and 1492R (5′-GGYTACCTTGTTACGACTT-3′). The reported 16S rRNA gene sequences were compared with reference sequences using the Basic Local Alignment Search Tool (BLAST).

### 4.5% (vol/vol) C_2_H_6_ addition under varying CH_4_ and/or O_2_ availabilities

To assess the impact of C_2_H_6_ addition alongside CH_4_ on the growth and PHB synthesis of *M. trichosporium* OB3b, four experimental sets (SET I, II, III, and IV) were designed under varying CH_4_ and O_2_ availabilities (vol/vol). SET I and SET III were conducted under sufficient O_2_ (30%), with CH_4_ supplied at 20% (SET I) or 10% (SET III). SET II and SET IV were conducted under limited O_2_ (5.5%), with CH_4_ supplied at 20% (SET II) or 10% (SET IV). Each set included two conditions: a 4.5% (vol/vol) C_2_H_6_-added treatment and a pure CH_4_ control, where 4.5% (vol/vol) Ar replaced C_2_H_6_. This C_2_H_6_ concentration (4.5%) was selected to reflect the typical range of C_2_H_6_ found in natural gas (0–15%) and serve as a baseline for subsequent experiments with increasing C_2_H_6_ concentrations. Atmospheric pressure was maintained using Ar. This design resulted in eight distinct headspace conditions (four sets, two conditions per set), as summarized in Fig. 1. Precultures of *M. trichosporium* OB3b, grown in 20% CH_4_ with air, were inoculated, resulting in an initial optical density (OD_600_) of 0.08–0.10. After the 48 h nutrient-balanced growth phase, the PHB accumulation phase was performed by resetting the headspace to the initial conditions applied during the preceding growth phase.

### Analyzing the effects of diverse concentrations of C_2_H_6_ under SET III

To explore the effects of varying C_2_H_6_ concentrations under specific CH_4_ and/or O_2_ availability, SET III (10% CH_4_ with 30% O_2_) was selected. This choice was based on the observation that 4.5% C_2_H_6_ addition in SET I–SET IV yielded the most significant results in SET III. Five different C_2_H_6_ concentrations were tested: “E0” (0% C_2_H_6_), “E5” (5% C_2_H_6_), “E10” (10% C_2_H_6_), “E20” (20% C_2_H_6_), and “E50” (50% C_2_H_6_). To confirm the obligate methanotrophy of *M. trichosporium* OB3b, an additional condition, “ObliE20” (20% C_2_H_6_ as the sole carbon source), was included. The atmospheric pressure in all conditions was balanced using Ar. For reproducibility, E0 with the same composition as the pure CH_4_ control in the preceding SET III was reiterated. The experimental setups are outlined in Fig. 1. Precultures grown in 20% CH_4_ with air were inoculated (initial OD_600_ = 0.08–0.10). Following the 48 h nutrient-balanced growth phase, PHB accumulation was evaluated under headspace conditions that were freshly reset to match the initial gas composition used during the growth phase. All experiments were conducted in triplicate.

### Three hypotheses tests

For hypotheses tests to elucidate the effects of C_2_H_6_, additional experimental setups—FE20, FobliE20, ME20, and AC20—were constructed by modifying the conditions of E20 (10% CH_4_, 30% O_2_, and 20% C_2_H_6_) and ObliE20 (30% O_2_, 20% C_2_H_6_). Specifically, 8.85 mM methanol was supplied in the ME20 medium, whereas 8.85 mM sodium formate was added to the FE20 and FobliE20 media. The AC20 medium contained 17.7 mM sodium acetate. These methanol and formate concentrations correspond to the stoichiometric oxidation of 10% CH_4_, whereas the acetate concentration assumes the complete oxidation of 20% C_2_H_6_. Precultures of *M. trichosporium* OB3b grown in 20% CH_4_ with air were employed as inoculum. The nutrient-balanced growth phase lasted 48 h, followed by the PHB accumulation phase under the same conditions. All experiments were conducted in triplicate.

### Quantitative real-time reverse transcription PCR (RT-qPCR)

*M. trichosporium* OB3b cultures (3 mL) for RT-qPCR analysis were sampled twice during cultivation under E0 and E20 conditions: during the mid-to-late exponential growth phase and during the PHB accumulation phase under E0 and E20 conditions (Fig. S6). Extracted and centrifuged cells were sent to Ebiogen Inc. (Seoul, Republic of Korea) for RNA extraction (using TRIzol reagent), purification, reverse transcription (cDNA synthesis), and RT-qPCR. For cDNA synthesis, 100 ng of total RNA was reverse-transcribed using SuperScript II Reverse Transcriptase (Invitrogen, Carlsbad, CA). RT-qPCR was conducted to quantify the relative expression levels of *pmoA* (encoding a subunit of pMMO) and *mmoX* (encoding a subunit of sMMO) genes in *M. trichosporium* OB3b cultivated in the presence (E20) or absence (E0) of C_2_H_6_ under both nutrient-sufficient growth and nutrient-imbalanced PHB accumulation phases. Gene-specific primers used in this study are listed in Table 5. The reactions were performed on 96-well PCR plates using the 2× Power SYBR Green Master Mix (Applied Biosystems, Foster City, CA, USA) on a QuantStudio 1 Real-Time PCR system (Applied Biosystems, Foster City, CA, USA).

**TABLE 5:** Gene-specific primers used in this study

Target gene	Primer	Sequence (5′–3′)	References
*pmoA*	*pmoA*_For	TTCTGGGGCTGGACCTAYTTC	(43, 83, 85, 86)
	*pmoA*_Rev	CCGACAGCAGCAGGATGATG	
*mmoX*	*mmoX*_For	TCAACACCGATCTSAACAACG	(43, 83, 85, 86)
	*mmoX*_Rev	TCCAGATTCCRCCCCAATCC	
16S rRNA	16S rRNA_For	GCAGAACCTTACCAGCTTTTGAC	(85–87)
	16S rRNA_Rev	CCCTTGCGGGAAGGAAGTC	

The three-step thermal cycle program included an initial denaturation at 95°C for 10 min, followed by 40 cycles of denaturation (95°C for 30 s), annealing (58°C for 20 s), and extension (68°C for 30 s). After the completion of amplification cycles, melting curve analyses (55°C–95°C) were carried out to confirm the specificity of the PCR products. Relative expression of the target genes was quantified using the *2*^*−ΔΔCt*^ method (88), with 16S rRNA as the reference gene for normalization (83, 85–87, 89–91) (File S3).

### Kinetic analyses

Kinetic parameters of growing cells, including the maximum specific CH_4_ utilization rate (*q_max_CH4_*, mg CH_4_·mg TSS^−1^·h^−1^), maximum specific O_2_ utilization rate (*q_max_O2_*, mg O_2_·mg TSS^−1^·h^−1^), and maximum specific C_2_H_6_ utilization rate (*q_max_C2H6_*, mg C_2_H_6_·mg TSS^−1^·h^−1^), were calculated according to the previous studies (45, 82, 92) (Files S1 and S2). Specifically, for example, in batch systems, the mass balance for CH_4_ consumption can be expressed as:


(eq. 7) $$\frac{-dM_{S}}{dt}=q_{max-CH4}XV_{L}$$

(eq. 8) $$dM_{S}=dC_{G-CH4}V_{G}+dC_{L-CH4}V_{L}$$

where *dM_S_* means the mass change in CH_4_; *C_G_* and *C_L_* denote the concentrations (mg/L) of CH_4_ in the gas and liquid phases, respectively, and *V_G_* and *V_L_* indicate the volumes of the gas and liquid (L), respectively. By using the dimensionless Henry’s law constant for CH_4_ (*H_C_CH4_*), equation 8 can be reformulated as equation 9: the applied Henry’s law constants and temperature dependence constants are 0.0014   mol·kg^−1^·atm^−1^ with 1,600 K for CH_4_, 0.0013   mol·kg^−1^·atm^−1^ with 1500 K for O_2_, and 0.0019   mol·kg^−1^·atm^−1^ with 2400 K for C_2_H_6_ (92, 93). These values were temperature-corrected (303.15 K) using respective temperature dependence constants and subsequently converted to dimensionless form (*C_G_/C_L_*), yielding *H_C-CH4_* of 31.4, *H_C-O2_* of 33.6, and *H_C-C2H6_* of 24.1 (Files S1 and S2).


(eq. 9) $$\frac{dC_{L-CH4}}{dt}=-\frac{q_{max-CH4}XV_{L}}{H_{C-CH4}V_{G}+V_{L}}$$

Experimental values of *dC_L_/dt* and biomass (TSS) concentration (*X*) at each time point obtained during the exponential growth phase were used to calculate *q_max-CH4_* through the nonlinear least-squared fitting, minimizing discrepancies between the experimental and model-predicted data (92). The sample method described above was identically applied to calculate *q_max_O2_* and *q_max_C2H6_*.

The maximum specific growth rate (*µ_max_*, h^−1^) was determined from the slope of the natural log of TSS against time, that is, $\mu_{max}=\frac{dln\left(TSS\right)}{dt}$, using data collected from the exponential growth phases where *q_max_* values were also derived (94). At least three data points from the exponential growth phase were utilized to calculate the aforementioned parameters. Detailed raw data and kinetic analyses are provided in Files S1 and S2.

### Analytical methods

For monitoring the growth of *M. trichosporium* OB3b, cell biomass was measured in terms of optical density (OD_600_) and total suspended solids (TSS, mg/L). OD_600_ was measured at 600 nm using a UV-Vis spectrophotometer (Thermo Scientific, Genesys-180, USA). To calculate TSS from OD values, the OD-TSS correlation equation obtained in our previous study was used: TSS = 811.45 * OD + 21.981 (R^2^ = 0.994) (82). In brief, standard cell culture samples (0.5–4.0 mL) with known OD values were vacuum-filtered through pre-washed, pre-dried, and pre-weighed 0.20 µm membrane filters (HYUNDAI MICRO Co., Ltd., Seoul, Republic of Korea). The filtered cell biomass and membrane filters were dried at 105°C for 24 h and then weighed (82, 95). The plotted TSS data of standard samples were fitted to a linear model. During the growth phase, OD values were periodically measured and converted to TSS using the aforementioned OD-TSS correlation equation.

Gas concentrations of CH_4_, O_2_, CO_2_, and C_2_H_6_ during the nutrient-balanced growth phase were analyzed using an Agilent 8890 gas chromatography (GC) system equipped with a thermal conductivity detector (TCD), MolSieve 5A column, and Porapak Q column. Headspace samples (0.1 mL) from each serum bottle were periodically withdrawn using a gastight syringe (Hamilton, Bonaduz, Switzerland) and manually injected into the GC inlet. The following parameters were used: injector, 150°C; oven, 105°C; and TCD, 200°C. Ar was used as the carrier gas. Peak areas of CH_4_, O_2_, CO_2_, and C_2_H_6_ were compared to standards and quantified.

Methanol consumption was measured by collecting 1 mL cultures immediately after inoculation and after 48 h during the nutrient-balanced growth phase. Samples were filtered through a 0.20 µm filter and stored at −20°C. Methanol concentration in the medium was analyzed using an Agilent 8890 GC system coupled with an Agilent 5977B mass spectrometer and an Agilent 7697A headspace sampler. Separation was performed on a DB-WAX column under the following conditions: oven, ramped from 50°C to 80°C at 5°C/min; inlet, 250°C; detector, 280°C; headspace oven, 70°C; loop, 80°C; transfer line, 90°C. A 0.1 mL headspace sample was injected for each analysis. Methanol was quantified by comparing peak areas to those of external standards.

To confirm acetate generation via C_2_H_6_ oxidation, 1 mL cultures were collected at the beginning and end of the PHB accumulation phase, filtered through a 0.20 µm filter, and stored at −20°C. Acetate concentration in the medium was measured using a Shimadzu GC-2010 Plus equipped with a flame ionization detector (FID) and an HP-INNOWAX column. The injector and detector were maintained at 280°C. The oven temperature was held at 100°C for 3 min, ramped to 250°C at 25°C/min, and held for 5 min. Nitrogen was used as the carrier gas, and nitrogen, hydrogen, and air were supplied as auxiliary gases. A 2  µL aliquot was injected, and acetate was quantified by comparing peak areas with those of external standards.

PHB content was quantified following the protocol outlined previously (51, 82, 96). At the end of the PHB accumulation phase, cell cultures were harvested, centrifuged twice at 3,500 rpm for 20 min, and the biomass pellet was freeze-dried for 24 h. The dried pellet was weighed and transferred to a 12 mL glass vial, which was then amended with 2 mL chloroform and 2 mL acidified methanol (3% sulfuric acid, 0.25 mg/mL benzoic acid). The vials were sealed, shaken, and heated at 100°C for 3.5 h. After cooling, 1 mL deionized water was added to separate the aqueous and organic phases. The organic phase was analyzed using an Agilent 8890 GC with an HP-5 column and a FID. The oven temperature program was as follows: 160°C for 4 min, 200°C for 4 min, and 275°C for 6 min. DL-3-hydroxybutyric acid sodium salt was used as an external standard. PHB content (wt%, w_PHB_/w_CDW_) was calculated by normalizing the PHB mass to the initial cell dry weight (CDW).

### Statistical analyses

Mean values and standard deviations were calculated from triplicate cultures for all experimental conditions. Linear and non-linear relationships between two variables were analyzed through linear/non-linear curve fitting using MATLAB, with the fit quality evaluated by the coefficient of determination (R^2^). Comparisons of specific experimental conditions (e.g., 4.5% C_2_H_6_ treatment) with their corresponding controls were performed using one-way analysis of variance (ANOVA) to identify significant differences. All *P-*value computations for two-tailed *t*-tests and ANOVA were conducted using Excel software.

## Data Availability

All data generated and analyzed during the current study are available from the corresponding author on reasonable request.

## SUPPLEMENTAL MATERIAL

File S1 aem.00969-25-s0001.xlsx Detailed raw data and kinetic analyses associated with Table 1 and Fig. 2.

File S2 aem.00969-25-s0002.xlsx Detailed raw data and kinetic analyses associated with Table 2 and Fig. 4.

File S3 aem.00969-25-s0003.xlsx Detailed raw data of RT-qPCR results associated with Fig. 7.

Supplemental material aem.00969-25-s0004.docx Tables S1 and S2; Figures S1 to S6.

ASM does not own the copyrights to Supplemental Material that may be linked to, or accessed through, an article. The authors have granted ASM a non-exclusive, world-wide license to publish the Supplemental Material files. Please contact the corresponding author directly for reuse.
