# Supplementary material for: Non-growth substrate ethane perturbs core methanotrophy in obligate methanotroph Methylosinus trichosporium OB3b upon nutrient availability
Source: Appl Environ Microbiol. 2025 Jul 10;91(8):e00969-25. doi: 10.1128/aem.00969-25 (PMC12366354; doi:10.1128/aem.00969-25)
Supplement: Supplemental material — Tables S1 and S2; Figures S1 to S6. [file aem.00969-25-s0004.docx]

**Non-growth substrate ethane perturbs core methanotrophy in obligate methanotroph *Methylosinus trichosporium* OB3b upon nutrient availability**

**SUPPLEMENTARY TABLES AND FIGURES**

**TABLE S1.** Methanol consumption in ME20 within 48 hours of the nutrient-balanced growth phase.

| Sample codes | Initial methanol (ppm) | Final methanol (ppm) |
| --- | --- | --- |
| ME20_#1 | 255.3 | Below detection level |
| ME20_#2 | 245.8 | 12.3 |
| ME20_#3 | 250.6 | 24.8 |

#1, #2, and #3 represent triplicate cultures of *Methylosinus trichosporium* OB3b. ME20 contained 8.85 mM methanol as an external reducing power and carbon source under the E20 condition—10% CH_4_, 30% O_2_, and 20% C_2_H_6_.

**TABLE S2.** Acetate generation in E20 during the PHB accumulation phase.

| Sample codes | Initial acetate (mg/L) | Final acetate (mg/L) |
| --- | --- | --- |
| E20 #1 | 12.5 | 59.20 |
| E20 #2 | 12.9 | 47.94 |
| E20 #3 | 14.2 | 55.44 |

#1, #2, and #3 represent triplicate cultures of *M. trichosporium* OB3b. E20 contained 10% CH_4_, 30% O_2_, and 20% C_2_H_6_.

**
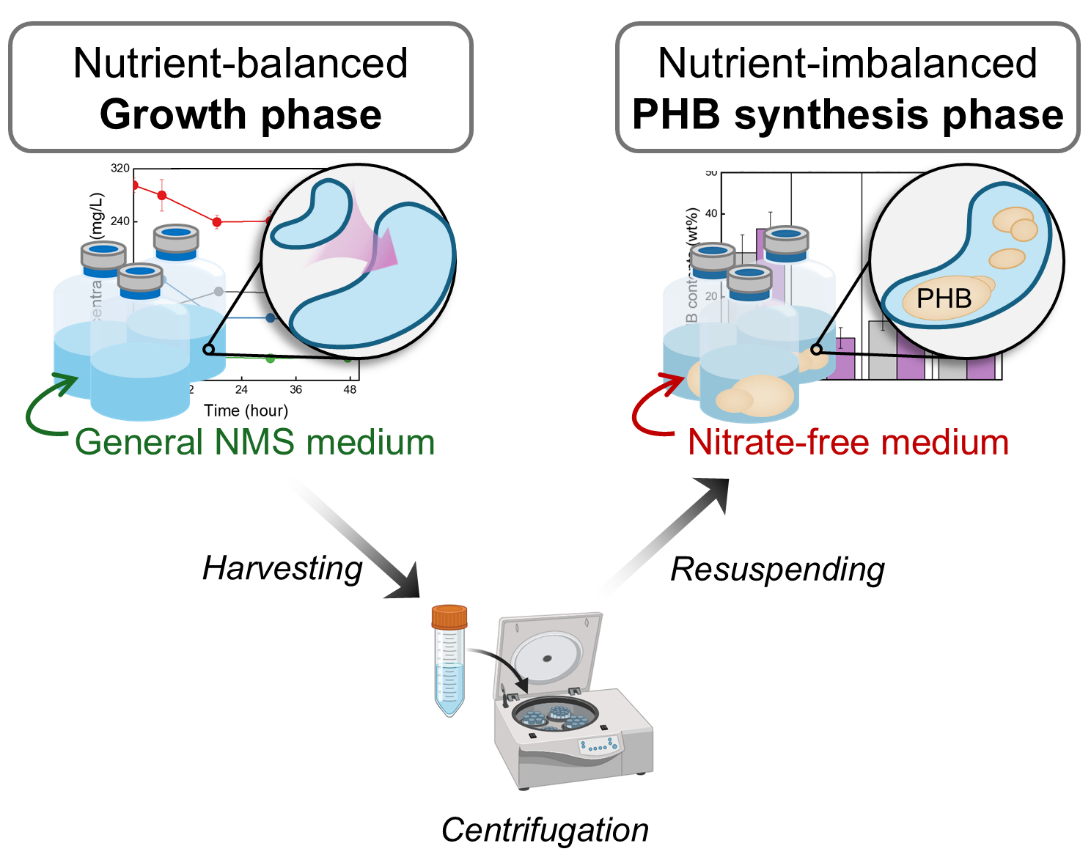
**

**FIG. S1**. Two-step cultivation of *Methylosinus trichosporium* OB3b. After the nutrient-balanced growth phase in nitrate salt medium (NMS) containing all necessary nutrients for cell growth, whole cell cultures were harvested and centrifuged (3,500 rpm, 20 minutes). The supernatant was discarded, and 5 mL was reserved. The cultures were then vortexed and resuspended in fresh medium, which did not contain a nitrogen source (nitrate) to induce PHB accumulation.

*
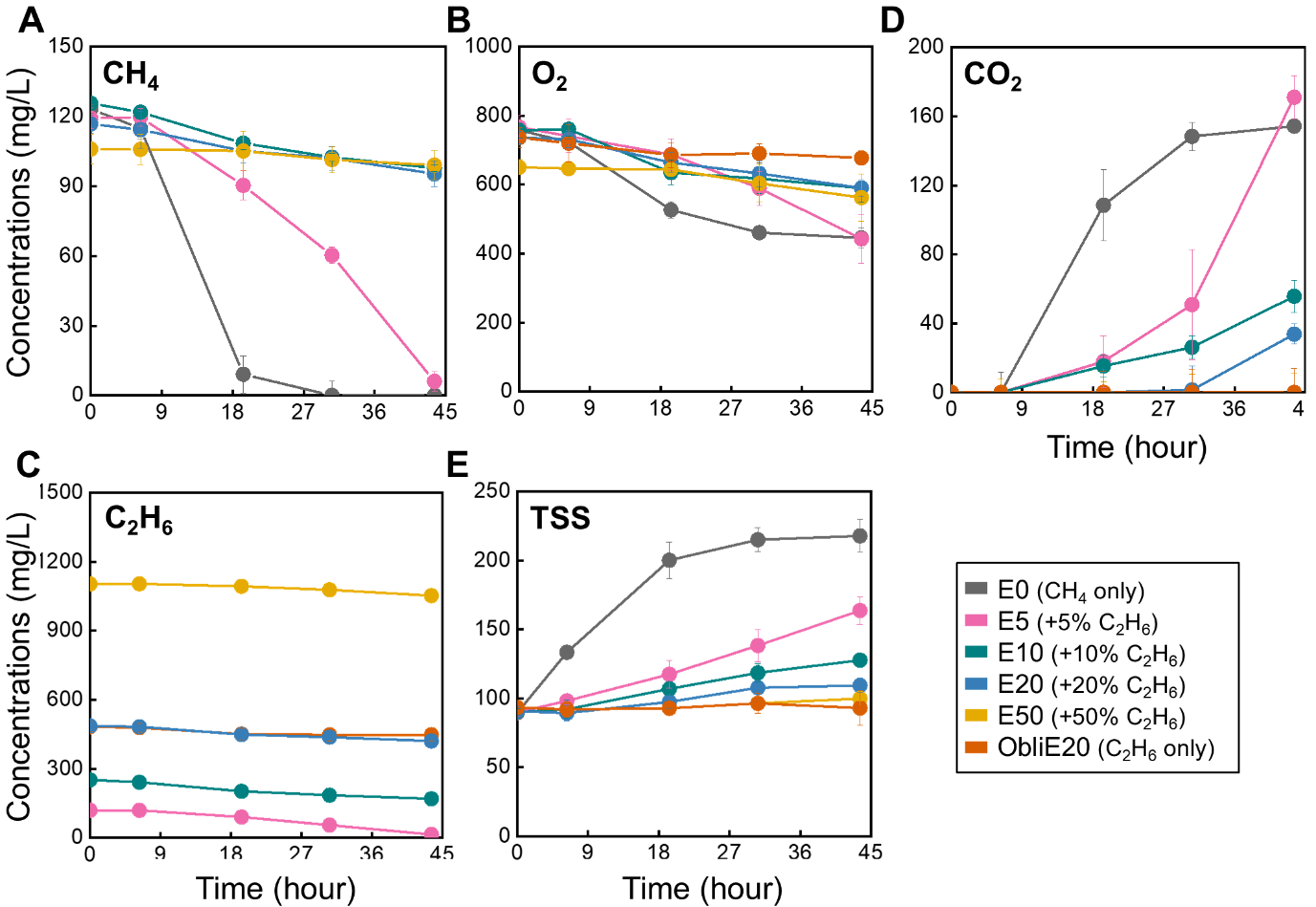
*

**FIG. S2.** Activity of *M. trichosporium* OB3b cultures with CH_4_ or CH_4_ with varying concentrations of C_2_H_6_ during the 45-hour growth phase. Panels show consumption of CH_4_ **(A)**, O_2_ **(B)**, and C_2_H_6_ **(D)**, and cumulative production of CO_2_ **(C)** and total suspended solids (TSS) **(E)**. Gray, pink, cyan, blue, yellow, and orange lines represent E0, E5, E10, E20, E50, and ObliE20 conditions, respectively. All conditions, except ObliE20, were supplied with 10% CH_4_ and 30% O_2_ (*v/v*), with C_2_H_6_ added at 0, 5, 10, 20, or 50% (E0 to E50). ObliE20 contained 20% C_2_H_6_ and 30% O_2_, without CH_4_. Values on the Y-axis indicate nominal concentrations, calculated per 0.05 L of liquid-phase volume. All experiments were performed in triplicate, and results are reported as mean ± standard deviation (SD).


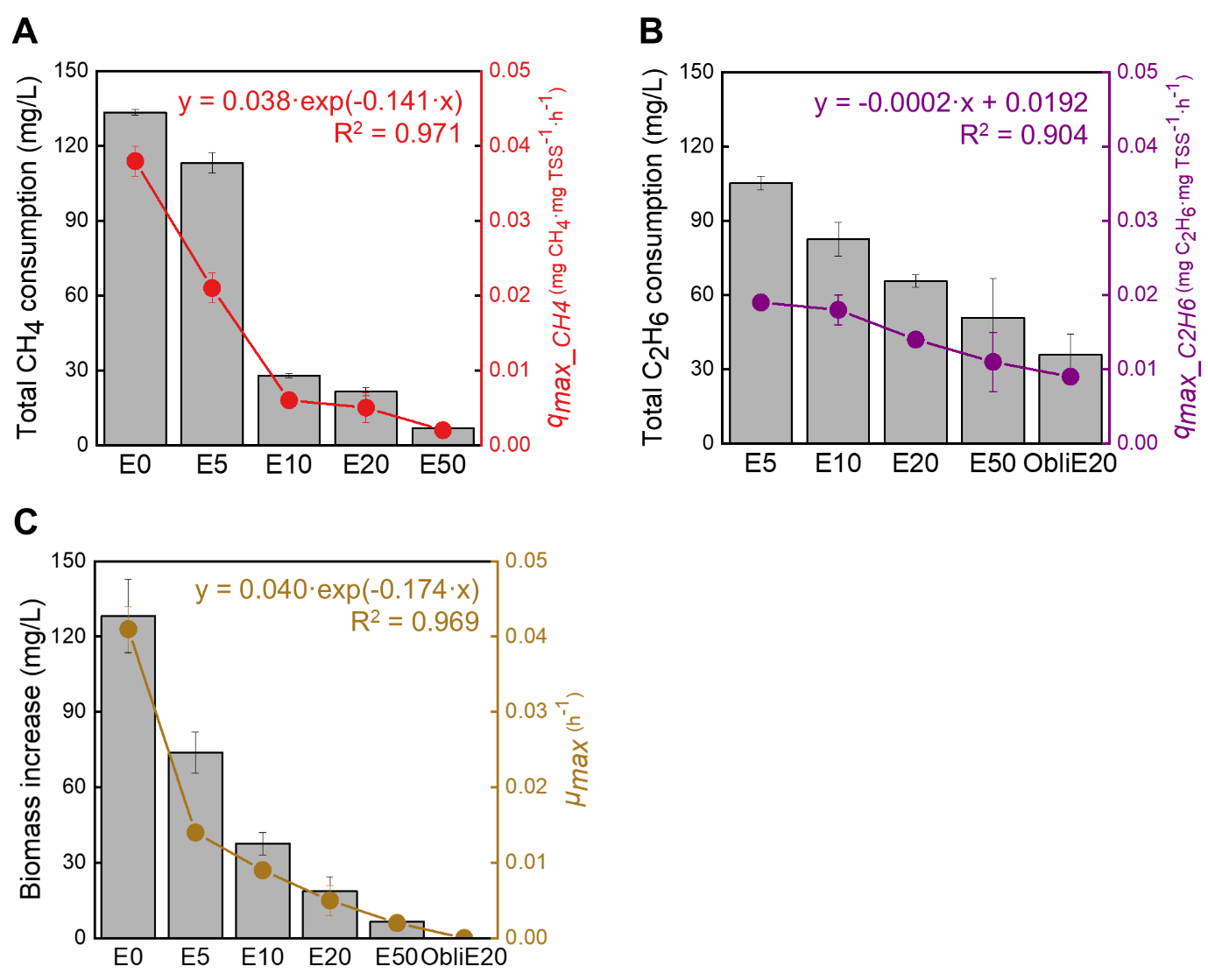


**FIG. S3**. Total consumption and maximum specific utilization rates (*q_max_*) of CH_4_ **(A)** and C_2_H_6_ **(B)**, and maximum specific growth rate (*µ_max_*) **(C)** of *M. trichosporium* OB3b cultured under different concentrations of C_2_H_6_ during the nutrient-balanced growth phases. Gray bars represent the total amount of gas consumed or biomass produced (nominal concentration), while red **(A)**, purple **(B)** brown **(B)** dots represent *q_max_CH4_*, *q_max_C2H6_*, and *µ_max_* under each condition, respectively. All experiments were carried out in triplicate, and data are presented as mean ± SD.


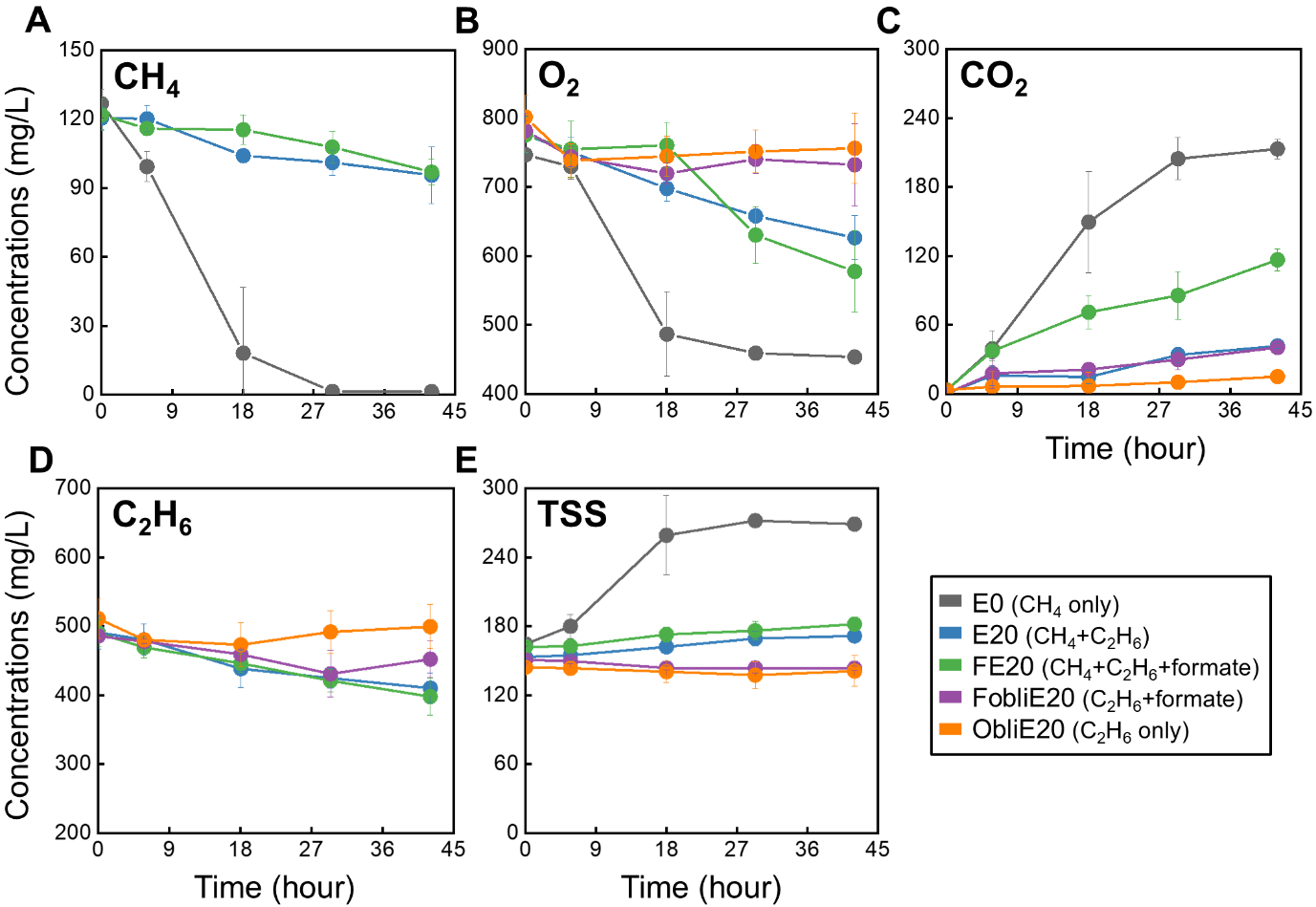


**FIG. S4.** Consumption of CH_4_ **(A)**, O_2_ **(B)**, and C_2_H_6_ **(D)**, and production of CO_2_ **(C)** and TSS **(E)** during the 45-hour growth phase by *M. trichosporium* OB3b. Gray, blue, green, purple, and orange lines represent the E0, E20, FE20, FobliE20, and ObliE20 conditions, respectively. All conditions included 30% O_2_ and 20% C_2_H_6_ (*v/v*); 10% CH_4_ was supplied only in E0, E20, and FE20, and omitted in FobliE20 and ObliE20. Sodium formate (8.85 mM) was added in FE20 and FobliE20. Concentrations on the Y-axis are expressed as nominal aqueous-phase values, derived by normalizing the total mass to 0.05 L of liquid volume. All experiments were performed in triplicate, and data are expressed as mean ± SD.


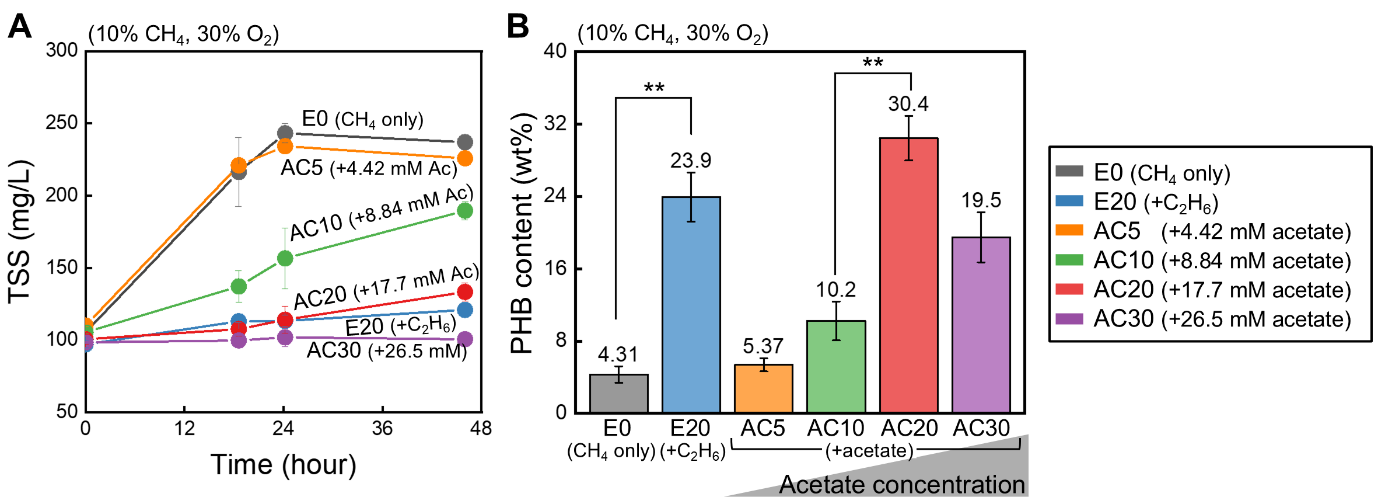


**FIG. S5.** Biomass production during the 48-hour growth phase **(A)** and PHB content after the subsequent 48-hour accumulation phase **(B)** at various acetate concentrations. Gray and blue data represent E0 and E20 conditions, respectively, both containing 10% CH_4_ and 30% O_2_ (*v/v*), with (E0) or without (E20) 20% C_2_H_6_. Sodium acetate was added to E0 at concentrations of 4.42, 8.84, 17.7, and 26.5 mM, corresponding to the theoretical oxidation products of 5, 10, 20, and 30% C_2_H_6_, and designated as AC5, AC10, AC20, and AC30, respectively. All experiments were conducted in triplicate, and results are expressed as mean ± SD.


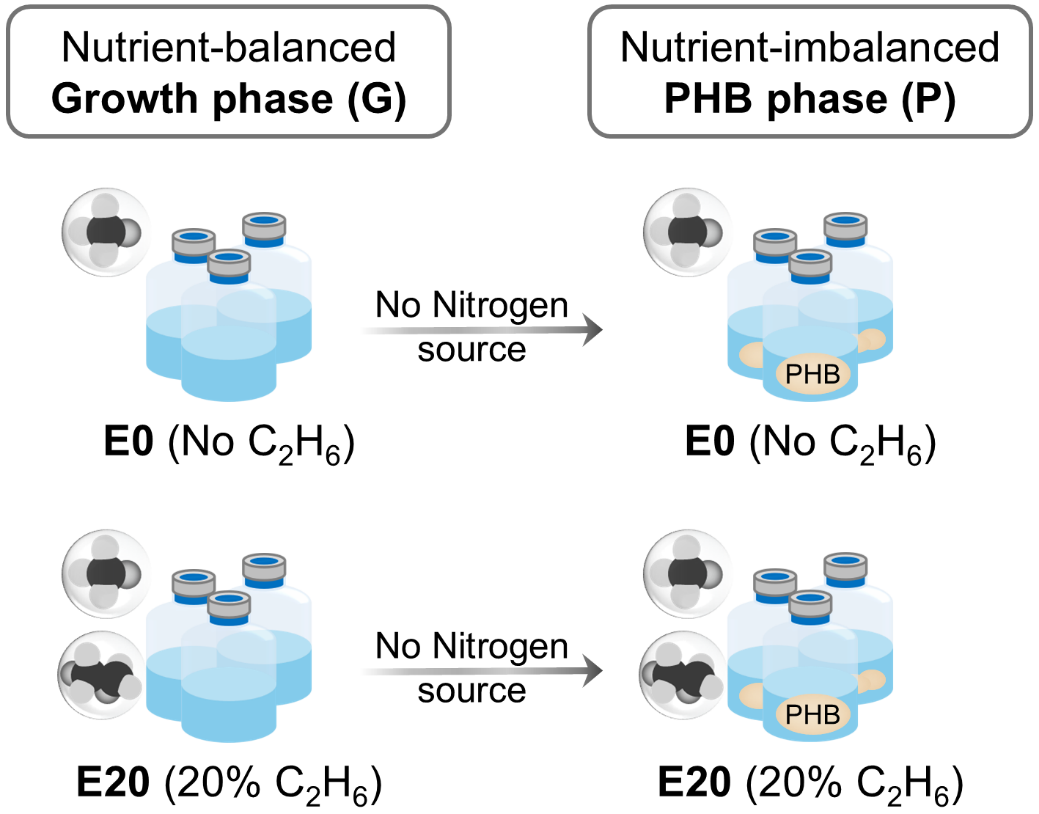


**FIG. S6**. Cultivation process of the samples used for RT-qPCR analysis. The triplicate cultures were grown under two conditions: E0 (without C_2_H_6_) and E20 (with 20% C_2_H_6_), following a two-stage cultivation process (growth phase and PHB accumulation phase). E0 and E20 samples for RT-qPCR analysis were collected at each stage. In the growth phase, sampling occurred during the mid-to-late exponential growth period, while in the PHB accumulation phase, samples were collected at the same time points as in the growth phase.
